# Cancer cells sense solid stress to enhance metastasis by CKAP4 phase separation-mediated microtubule branching

**DOI:** 10.1038/s41421-024-00737-1

**Published:** 2024-11-12

**Authors:** Xing Sun, Yangyang Zhou, Shengjie Sun, Siyuan Qiu, Menglan Peng, Han Gong, Junxiao Guo, Chengcai Wen, Yibin Zhang, Yifang Xie, Hui Li, Long Liang, Guoyan Luo, Wencan Wu, Jing Liu, Weihong Tan, Mao Ye

**Affiliations:** 1 Department of Hematology, the Second Xiangya Hospital, Molecular Biology Research Center, Center for Medical Genetics, School of Life Sciences, Hunan Province Key Laboratory of Basic and Applied Hematology, Central South University, Changsha, Hunan China; 2 Molecular Science and Biomedicine Laboratory, State Key Laboratory of Chemo/Biosensing and Chemometrics, College of Biology, College of Chemistry and Chemical Engineering, Aptamer Engineering Center of Hunan Province, Hunan University, Changsha, Hunan China; 3 Department of Dermatology, Hunan Engineering Research Center of Skin Health and Disease, Hunan Key Laboratory of Skin Cancer and Psoriasis, Xiangya Hospital, Central South University, Changsha, Hunan China; 4 Hangzhou Institute of Medicine (HIM), The Chinese Academy of Sciences, Hangzhou, Zhejiang China; 5 The Eye Hospital of Wenzhou Medical University, Wenzhou, Zhejiang China; 6 Oujiang Laboratory (Zhejiang Lab for Regenerative Medicine, Vision and Brain Health), Wenzhou, Zhejiang China; 7 Institute of Molecular Medicine (IMM), Renji Hospital, Shanghai Jiao Tong University School of Medicine, and College of Chemistry and Chemical Engineering, Shanghai Jiao Tong University, Shanghai, China

**Keywords:** Metastasis, Extracellular matrix, Cancer microenvironment

## Abstract

Solid stress, originating from rigid and elastic components of extracellular matrix and cells, is a typical physical hallmark of tumors. Mounting evidence indicates that elevated solid stress drives metastasis and affects prognosis. However, the molecular mechanism of how cancer cells sense solid stress, thereby exacerbating malignancy, remains elusive. In this study, our clinical data suggest that elevated stress in metastatic solid tumors is highly associated with the expression of cytoskeleton-associated protein 4 (CKAP4). Intriguingly, CKAP4, as a sensitive intracellular mechanosensor, responds specifically to solid stress in a subset of studied tumor micro-environmental elements through liquid–liquid phase separation. These micron-scaled CKAP4 puncta adhere tightly onto microtubules and dramatically reorchestrate their curvature and branching to enhance cell spreading, which, as a result, boosts cancer cell motility and facilitates distant metastasis in vivo. Mechanistically, the intrinsically disordered region 1 (IDR1) of CKAP4 binds to microtubules, while IDR2 governs phase separation due to the Ca_v_1.2-dependent calcium influx, which collectively remodels microtubules. These findings reveal an unprecedented mechanism of how cancer cells sense solid stress for cancer malignancy and bridge the gap between cancer physics and cancer cell biology.

## Introduction

Tumors, from a biophysical perspective, are elastic solids^1^. Their increase in size and reconstruction of the intratumor microenvironment gives rise to various physical abnormalities^2^. Solid stress, as a mechanical force present within and transmitted by solid and elastic components of the extracellular matrix and cells, becomes one of four physical hallmarks of tumor^3^. In recent studies, elevated solid stress has been quantitively mapped across many solid tumors^4,5^. Rather than a mere byproduct of tumor fibrosis, angiogenesis, and outgrowth, it actively initiates tumorigenesis, drives metastasis, and affects prognosis, owing to the activation of downstream cancerous signaling axes, including Wnt/β-catenin and MAPK-JNK/c-Jun^5–11^. However, the underlying molecular mechanism of how cancer cells sense and rapidly respond to solid stress, thereby exacerbating their malignant behaviors, remains elusive. Decoding their physical traits and underlying molecular basis furthers the understanding of cancer malignancy.

Biomolecular condensates, formed through a process called liquid–liquid phase separation (LLPS), are a class of functional supramolecular assemblies, which show remarkable abilities to buffer extracellular stresses, such as heat^12^, pH^13^, osmosis^14^, viral infections^15^, and mechanical perturbations^16–18^. In the mechanical scenarios, e.g., cardiac fibrosis^16^, niche stiffness sustained cancer stemness^19^, and cell–cell junctions^20^, LLPS plays an important role. Notably, scaled into micron size, these condensates can form robust mechanical platforms to promote cancer metastasis, for example, by activation of the EMT pathway or initiation of nuclear polarization^21–23^. As a challenge in multi-disciplinary fields, so far, the links between solid stress and phase separation, as well as their contribution to cancer metastasis is still an open question. Elucidating their association is a significant topic of broad concern.

Microtubules constitute the mechanical support of cell morphology, division, and migration, and represent one intracellular structure in driving cancer metastasis, during which microtubule nucleation, growth, stability, branching, and dynamics make great contributions. Biomolecular condensates have been reported to regulate these microtubule functions, for example, EB1 condensates to guide growth, Tau condensates for stability maintenance, and TPX2 droplets to nucleate branches^24–27^. In cancer metastasis, particularly in the mechanical context of solid stress, how microtubules function remains a field in infancy. Notably, as a backbone of cell architecture, how microtubule physics, i.e., geometry, bundling, and spreading, responds to extracellular mechanical stimuli is still poorly understood.

Cytoskeleton-associated protein 4 (CKAP4), or CLIMP63, mediates a variety of cancer signaling pathways^28–31^. Recently, it has attracted widespread attention as an endoplasmic reticulum (ER) residing protein, which shapes ER morphology^32^, indicates ER biology^33^ and regulates organelle distribution^34^. Furthermore, it can also regulate mitochondrial function by tuning the ER-mitochondria contact sites^35^. Posttranslational modifications (PTMs), mainly including palmitoylation, phosphorylation, and acetylation, play important roles in CKAP4 biological functions^36^. Considering the multiple biological functions and diverse roles in organelle-to-organelle communications, CKAP4 appears to be a universal organizer that coordinates intracellular events. One of our recent studies identified its ability to tune the stiffness gradient of cell membranes in cancer metastasis^37^, which hints at its association with cellular mechanical forces. However, whether and how CKAP4 senses and responds to mechanical stress remains to be determined.

In this study, we reported the crucial role of oncoprotein CKAP4 in bridging solid stress and cancer malignancy by LLPS. In this case, CKAP4 serves as a specific intracellular mechanosensor for solid stress, which may suggest an unprecedented paradigm of how solid stress is sensed by cancer cells. In function, these micron-scaled speckles adhere onto microtubules, and they are necessary and sufficient to mechanically direct microtubule spreading and branching both in vitro and in vivo. As a result, cancer cell motility is enhanced, which eventually leads to cancer metastasis. From a mechanistic insight, our data showed that CKAP4 LLPS was highly dependent on the robust interaction between the IDR2 domain and calcium ions, and condensates of small size exhibited more dramatic effects on microtubule modeling.

## Results

### CKAP4 is highly associated with elevated cell compaction in metastatic tumors

**Fig. 1:**
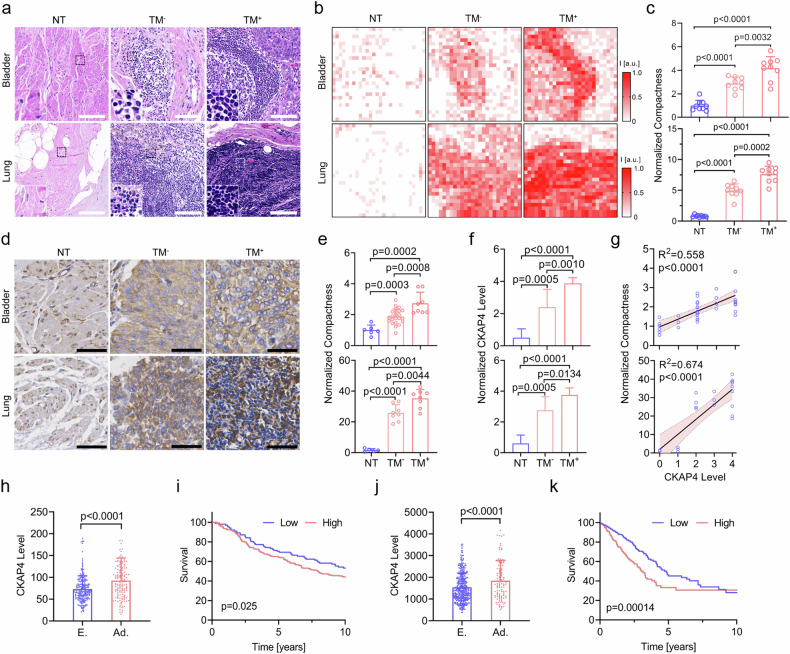
CKAP4 is highly associated with elevated cell compaction in bladder and lung cancers. **a** Hematoxylin and Eosin (H&E) staining of the bladder (top) or lung (bottom) normal tissues (NT, left), nonmetastatic tumor tissue (TM^−^, middle), and metastatic tumor tissue (TM^+^, right). Inset images showed enlarged details of the indicated areas. Scale bars, 100 μm. **b** Relative cell compactness of tissues in **a**, which was quantified by local cell density. **c** Quantification of compactness in the normal and tumor tissues. The top panel was data from BLCA, and the bottom was from LUAD. **d** IHC staining of CKAP4 in NT, TM^−^, TM^+^ tissues of BLCA and LUAD. CKAP4 was shown in brown. Scale bars, 100 μm. **e** Quantification of relative cell compactness in NT, TM^−^, and TM^+^ tissues of BLCA (top) and LUAD (bottom). **f** Quantification of relative CKAP4 expression in NT, TM^−^, and TM^+^ tissues of BLCA (top) and LUAD (bottom). **g** Association of CKAP4 expression and compactness in tumor tissues of BLCA (top) and LUAD (bottom) was evaluated by simple linear regression. **h** CKAP4 level of BLCA patients in early stage (E.) and advanced stage (Ad.). **i** Survival of BLCA patients in IMvigor210 cohort, as stratified by CKAP4 level. *P*-value was calculated by log-rank test. **j** CKAP4 level of LUAD patients in early stage (E.) and advanced stage (Ad.). **k** Survival of LUAD patients in TCGA database, stratified by CKAP4 level. *P*-value was calculated by log-rank test. In **c**, **e**, **f**, **h**, **j**, data were shown as mean ± SD and the *P*-values were calculated by Student’s *t*-test.

Cell compaction represents a dramatically reorchestrated mechanical landscape of tumor microenvironment (TME). To verify the association between cell compaction and cancer metastasis, we collected two types of representative solid tumor tissues, i.e., bladder urothelial carcinoma (BLCA) and lung adenocarcinoma (LUAD), and analyzed the cell compactness. Compared with the adjacent non-tumor (NT) sites, tumor sites in both BLCA and LUAD were subjected to dramatically elevated compactness, and particularly, tumors with metastasis (TM^+^) showed greater intercellular compaction than those without (TM^−^), as shown in Fig. 1a–c. Therefore, soared cell compactness was proved to be a typical trait of solid tumors in both BLCA and LUAD, and it is highly associated with tumor metastasis.

Next, to explore the role of cell compaction in driving tumor metastasis, we focused on a newly identified mechanically associated protein, CKAP4, which was discovered in one of our previous studies^37^. Firstly, immunohistochemistry (IHC) data showed that CKAP4 expression was positively correlated with elevated cell compactness in the TME, and it was significantly higher in staged solid tumor tissues (Fig. 1d–g). As shown in the database of The Cancer Genome Atlas Program (TCGA), CKAP4 overexpression was correlated with poor survival in both BLCA (Fig. 1h, i) and LUAD (Fig. 1j, k). By contrast, in the case of hematological cancers (e.g., acute myeloid leukemia (AML) and diffuse large B-cell lymphoma (DLBC)), free of compaction, CKAP4 expression was relatively low, and no significant correlation with survival was observed (Supplementary Fig. S1a–c). Consistently, western blotting analysis showed that CKAP4 was overexpressed in a broad subset of solid tumor cell lines, but not in leukemia cell lines (Supplementary Fig. S1d, e). Taken together, cell compaction is a typical physical trait in cancer progression, and CKAP4 may potentially bridge cell compaction and solid tumor metastasis.

### CKAP4 condensation sensitively responds to solid stress

**Fig. 2:**
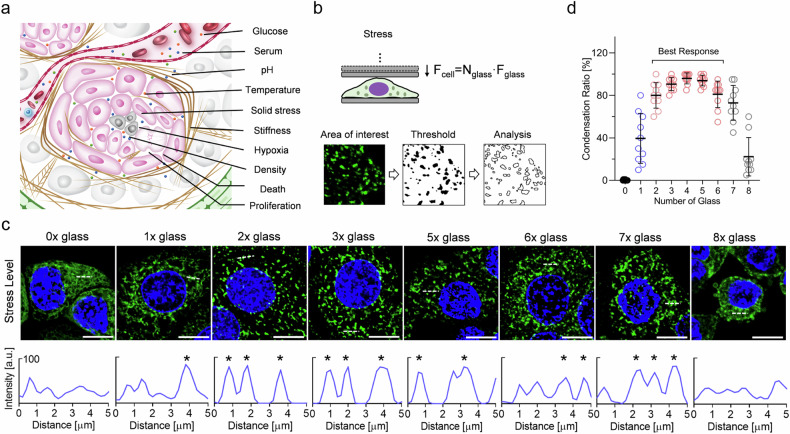
CKAP4 condensation responds sensitively to solid stress. **a** Schematic diagram of the characteristics of TME^3^. **b** Schematic diagram of the mechanical model at the cell level and used approach to analyze condensates. Different pieces of glass coverslips were used to adjust the magnitude of solid stress. **c** CKAP4 condensation responded to different levels of solid stress. The condensation level of CKAP4 was analyzed in the lower panel. Peaks of condensate were marked as asterisks. Scale bar, 10 μm. **d** The level of intracellular condensates in response to different levels of solid stress.

TME harbors a broad subset of characteristic traits^3^, such as pH, nutrition, temperature, solid stress, cell density, substrate stiffness, hypoxia, as well as increased proliferation, division, and cell death (Fig. 2a). Cell compaction is commonly confounded with these complex tumoral risk elements. To elucidate the function of CKAP4 in solid tumors, GFP-tagged CKAP4 was expressed in several commonly used cancer cell lines (i.e., 5637, TCC-SUP, Hela, and A549), and thin but rigid glass coverslips were used as a mechanical challenge of solid stress (Fig. 2b). Our data showed that CKAP4 in cancer cells started to condense in response to solid stress, and solid stress from 2 to 6 pieces of coverslips provided the best effects without obvious cell damage (Fig. 2c, d). Interestingly, concerning some other conceptually similar physical elements, i.e., cell density and substrate stiffness, neither was able to induce CKAP4 condensation (Supplementary Fig. S2a), such was also seen in the case of altered expression level, proliferation, death, and division (Supplementary Fig. S2b), as well as changes in glucose, serum, hypoxia, pH, and temperature (Supplementary Fig. S2c). Moreover, both endogenously- and exogenously-expressed CKAP4 showed consistent condensation across a broad subset of cancer cell lines (Supplementary Fig. S3a–c). GFP tag showed no obvious effect on CKAP4 condensation (Supplementary Fig. S3d, e). As indicated in Supplementary Fig. S3f–h, CKAP4 condensation was not due to stress-induced expression or artifacts from overexpression. Taken together, these data demonstrate that CKAP4 is potentially to be an important mechanosensor for solid stress.

### CKAP4 condensation requires constant and sufficient solid stress

**Fig. 3:**
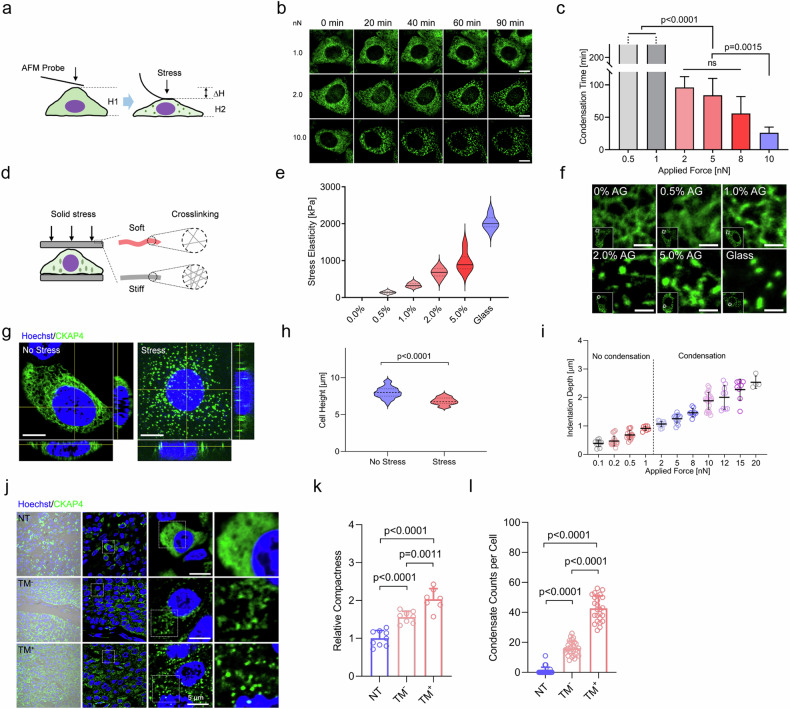
Solid stress promotes CKAP4 condensation in cell lines and tumor tissues from patients. **a** Schematic diagram of AFM-based strategy to investigate the force level that promotes CKAP4 condensation. **b** Representative images of CKAP4-GFP expressing 5637 cells compacted by AFM probes for CKAP4 condensation. CKAP4 was indicated by green color. Scale bars, 10 μm. **c** Average time from stress applied on cells to massive condensation of CKAP4. **d** Schematic diagram of stress solidity on CKAP4 condensation. AGs of various concentrations and different numbers of glass coverslips are used. Solidity is quantified by AFM-based material elasticity. **e** Elasticity of applied materials (AG and glass) was measured by AFM probe. For each material, three different positions were measured with three repetitions. **f** Representative confocal images of CKAP4 condensation under various solid stress of AGs and glass coverslips. CKAP4 was shown in green color. Scale bars, 5 μm. **g** Representative three views of confocal images of cells with or without stress. CKAP4 was shown in green, and Hoechst in blue. Scale bars, 10 μm. **h** Quantification of cell height with or without solid stress. **i** The association of stress level and cell height. **j** CKAP4 condensation in BLCA NT, TM^−^, and TM^+^ tissues. Nuclei were stained by Hoechst, and CKAP4 was stained by antibody (green color). Scale bars, 5 μm. **k** Quantification of relative compressive compactness in NT, TM^−^, TM^+^ tissues. **l** Quantification of relative CKAP4 condensation per cell in NT, TM^−^, and TM^+^ tissues. In **c**, **e**, **h**, **k**, **l**, data were represented as mean ± SD, and the *P*-values were calculated by Student’s *t*-test.

To accurately quantify the cell responsiveness to solid stress, two key physical parameters of solid stress, i.e., force magnitude and stress elasticity, were quantified, respectively. Firstly, atomic force microscopy (AFM) was used to measure the force applied on the cell surface, and the process of CKAP4 condensation was continuously tracked under confocal microscope (Fig. 3a). As shown in Fig. 3b, c, CKAP4 started to condense into small speckles when the applied force is greater than 2.0 nN, and CKAP4 condensation could be induced in less than 40 min as applied force is 10 nN. As the calculation was based on a classic physical model, solid stresses applied on cells by both coverslips and AFM probes were at a similar level (Supplementary Fig. S4a), and it was on a similar order of magnitude in tumor tissues as reported before (Supplementary Fig. S4b)^4,38,39^. By contrast, intermittent stress by AFM probes failed to induce condensation no matter how force was applied (Supplementary Fig. S5a, b).

Secondly, to investigate the effect of elasticity, agarose gels (AGs) of various crosslinking degrees were used. As shown in Fig. 3d, by controlling applied forces at identical levels, stress with various elasticity were exerted on cells. Our data showed that soft gels could not induce CKAP4 condensation, while those of high elasticity (1% AG, 338 ± 55 kPa) could (Fig. 3e, f). Notably, this elastic level is higher than those of normal tissues, but comparable to those of tumors^2^. Thus, the magnitude of force and elasticity of applied material are necessary to induce CKAP4 condensation in cancer cells, highlighting the dominant role of solid stress in driving CKAP4 condensation. We also noticed that around 1 μm of cell height was lowered under solid stress, while cell density or substrate stiffness did not have this effect (Fig. 3g–I; Supplementary Fig. S5c–f), discriminating the trait of solid stress from other physical forces.

To investigate the role CKAP4 condensation in cancer progression, 25 cases of BLCA and 21 cases of LUAD samples were collected, and CKAP4 was stained with antibodies in sectioned slices. In adjacent normal tissues, cells harbored dispersed CKAP4, but in tumor sites of great compaction CKAP4 was shown as speckles, and in metastatic cases (TM^+^), cancer cells exhibited more evident and denser CKAP4 speckles (Fig. 3j–l; Supplementary Fig. S5g, h), in line with the observation of CKAP4 speckles in the cell lines (Figs. 2c and 3g). Taken together, our data showed that CKAP4 could sense and respond to solid stress at both cell level and tissue level through molecular condensation, suggesting its importance in tumor progression.

### CKAP4 undergoes phase separation both in vivo and in vitro

**Fig. 4:**
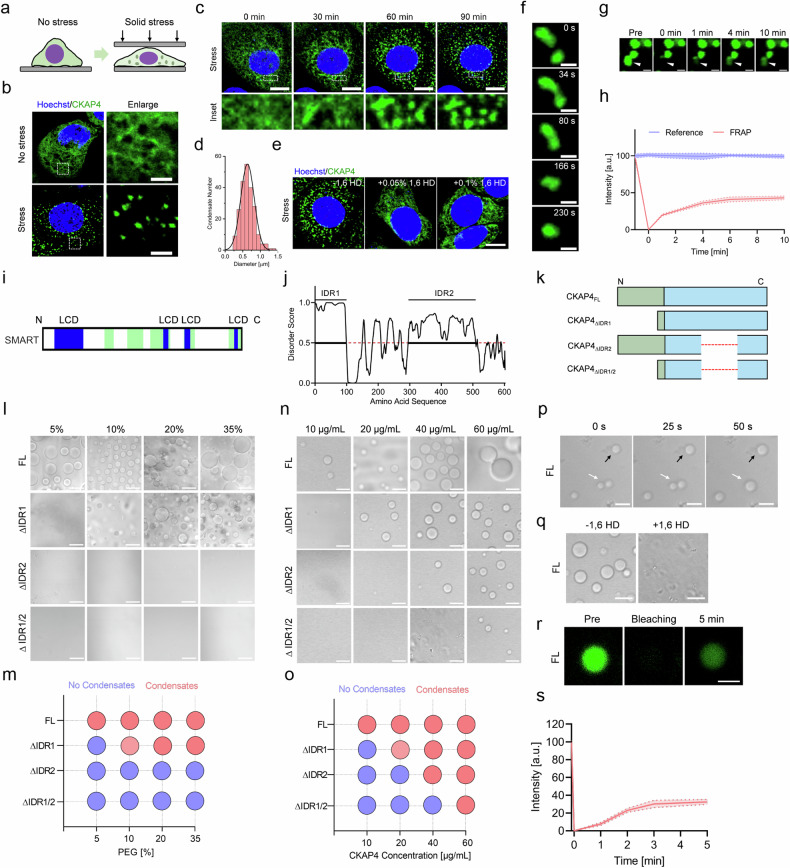
CKAP4 undergoes phase separation both in vivo and in vitro*.* **a** Schematic diagram of the mechanical model to investigate CKAP4 condensation in cancer cells. **b** Representative images of CKAP4-GFP-expressing 5637 cells with or without 3 h of solid stress. Cell nuclei were stained by Hoechst. Detailed CKAP4 expression in the indicated area in cells was shown in the right panel. Scale bars, 5 μm. **c** Time-lapse imaging of CKAP4 condensation in stressed CKAP4-GFP-expressing 5637 cells. Nuclei were stained with Hoechst. Enlarged CKAP4 condensation of the indicated areas was shown in the bottom panel. Scale bars, 10 μm. **d** Size distribution of CKAP4 condensates in cells. More than 100 condensates were calculated. **e** Inhibition of CKAP4 condensation with 0.05% and 0.1% of 1,6 HD for 2 min. Scale bar, 10 μm. **f** Time-lapse imaging of the fusion of CKAP4 condensates in cells under stress. Images were collected with a time interval of 30 s. Scale bars, 1 μm. **g**, **h** FRAP of CKAP4-GFP condensates in cells. Images were collected with a time interval of 1 min. Scale bars, 1 μm. **i** SMART analysis of LCDs in CKAP4. **j** PONDR analysis of IDRs in CKAP4. **k** Schematic of CKAP4 and truncated variants. **l**, **m** Phase separation of CKAP4 and the truncated variants in crowding reagent PEG 5000. Protein concentration was controlled at 20 μg/mL. Scale bars, 10 μm. **n**, **o** effect of protein concentration on the phase separation of CKAP4 and truncated variants. The concentration of PEG 5000 was 20% (w/v). Scale bars, 10 μm. **p** The fusion and growth of CKAP4 condensates. Images were collected with a time interval of 25 s. Scale bars, 10 μm. **q** Inhibition of CKAP4 phase separation by 1,6 HD. Scale bars,10 μm. **r**, **s** FRAP of CKAP4 condensates. CKAP4 was fluorescently labeled with a previously identified FITC aptamer. Images were collected with a time interval of 1 min. Scale bar, 10 μm.

To uncover the function of CKAP4 condensates, the physical trait of condensates was investigated. As shown in Fig. 4a–c, solid stress shifted dispersed CKAP4 into condensed speckles in less than 60 min. The size of speckles ranged from 0.2 to 1.5 μm, with an average size of 0.63 ± 0.19 μm (Fig. 4d). After treatment with 1,6-hexanediol (1,6 HD, an inhibitor for phase separation), intracellular CKAP4 were unable to condense into speckles (Fig. 4e). Moreover, CKAP4 speckles exhibited as a fluid, featured as frequent fusion (Fig. 4f) and fluorescence recovery after photobleaching (FRAP) was observed (Fig. 4g, h), consistent with the concept of phase separation.

To understand the characteristics of CKAP4 phase separation, the amino acid sequence was firstly analyzed. As shown in Fig. 4i, j, CKAP4 showed typical low complexity domains (LCDs) and intrinsically disordered regions (IDRs). The predominant overlapping of such regions may suggest the ability of CKAP4 in phase separation. To prove this, both full-length (FL) and IDR-truncated variants of CKAP4 were purified for testing in vitro (Fig. 4k). As presented, 20 μg/mL of CKAP4^FL^ could form evident droplets in buffer containing 5% PEG 5000. By contrast, the capacity of truncated CKAP4 toward phase separation was significantly attenuated in CKAP4 with ∆IDR1 which needed at least 10% PEG 5000 to condensate, while ∆IDR2 and ∆IDR1/2 almost lost the ability to condense completely (Fig. 4l, m). Similarly, when controlling the concentration of PEG 5000 at 20%, 10 μg/mL CKAP4^FL^ started to coalesce into droplets, while that of ∆IDR1 needed 20 μg/mL; ∆IDR2 and ∆IDR1/2 were even higher, requiring at least 40 μg/mL and 60 μg/mL, respectively (Fig. 4n, o). Thus, CKAP4^FL^ showed the ability to undergo phase separation, whereas truncated variants either depleted IDR1 or IDR2 showed mitigated ability, highlighting the significance of the IDR region. Same as other reported biomolecular condensates, CKAP4 condensates could fuse (Fig. 4p), be disrupted by 1,6 HD (Fig. 4q), and recover fluorescence after bleaching (Fig. 4r, s). Taken together, our data indicated that CKAP4 condensation followed the rule of phase separation, both in vitro and in vivo.

### CKAP4 condensation depends on Ca_v_1.2-mediated calcium influx, and IDR2 is the main region for Ca^2+^ interaction

**Fig. 5:**
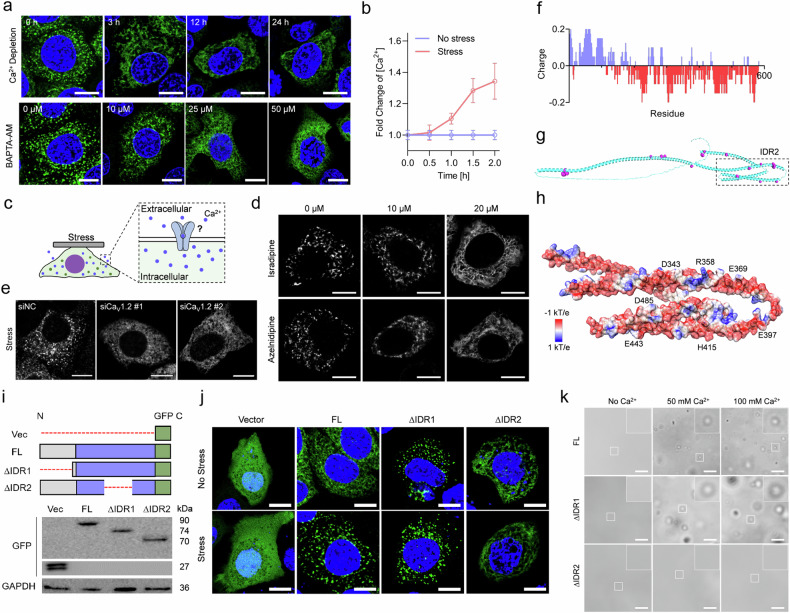
CKAP4 condensation depends on Ca_v_1.2-mediated calcium influx, and IDR2 is the main region for Ca^2+^ interaction. **a** Cells were treated with calcium-depleted (top panel) or calcium-chelated (by MAPTA-AM) culture medium for the indicated periods, and CKAP4 condensation in stressed cells was imaged under a confocal microscope. Scale bars,10 μm. **b** Intracellular calcium concentration in cells with or without solid stress is determined by Inductively ICP-OES. **c** Schematic of calcium ion-induced CKAP4 condensation. **d** Isradipine and azelnidipine were used to block the calcium channel. Representative images of CKAP4 condensation were shown. Scale bars, 10 μm. **e** After Ca_v_1.2 was knocked down, CKAP4 was not able to undergo phase separation under solid stress. Scale bars, 10 μm. **f** Charge distribution in CKAP4 was analyzed, setting the window of amino acid length as 10 amino acids. Positive charge was indicated by blue color, and negative charge by red. **g** Interaction between CKAP4 (green) and calcium ions (pink) was analyzed using the online program BioMetAll, and the structure of CKAP4 was simulated by AlphaFold2. Strong interaction in the IRD2 region was enlarged. **h** Surface charge of CKAP4 in IDR2, and potential binding sites of Ca^2+^. **i** Schematic diagram of proteins with/without IDR truncation, and western blotting analysis of GFP and GFP-fused CKAP4 and CKAP4 truncations were shown. **j** Representative images of 5637 cells under stress. GFP-fused proteins in (**h**) were expressed in cells. Scale bars, 10 μm. **k** Ca^2+^ promoted CKAP4 condensation in an IDR2-dependent manner. Scale bars, 5 μm.

To uncover how CKAP4 condensation was initiated under solid stress, a variety of classic mechanically associated pathways were investigated (Supplementary Fig. S6a). As shown in Supplementary Fig. S6b–d, neither inhibition of focal adhesion kinases (FAK) with PF-573228 nor mutation of phosphorylation and palmitoylation sites of CKAP4 was sufficient to reverse the state of CKAP4 in cells, suggesting no dramatic effects of such pathways. Considering that calcium ion-mediated biological processes are commonly fast-responding, and are associated with mechanical stress, we assumed calcium signaling might play a role in CKAP4 condensation. Indeed, once calcium was depleted from the culture medium, stressed cells were unable to induce CKAP4 condensation, and after chelation of intracellular calcium ion by 10 μM BAPTA-AM, CKAP4 also lost its condensation ability (Fig. 5a). As measured by Coupled Plasma Optical Emission Spectrometer (ICP-OES), intracellular Ca^2+^ was elevated under stress (Fig. 5b). To verify how calcium ions were modulated, a variety of agonists of calcium channels were examined (Supplementary Fig. S6e). In the absence of solid stress, the usage of agonists of the Ca_v_ family (Urolithin C or Bay-K-8644) but not those of the Piezo and TRP family was able to promote CKAP4 phase separation (Supplementary Fig. S6f). Noticing that both urolithin C and bay-K-8644 target L-type calcium channel Ca_v_1.2, we used two specific inhibitors, isradipine, and azelnidipine. Both of them could inhibit solid stress-induced phase separation of CKAP4 (Fig. 5c, d), which was further confirmed by Ca_v_1.2 knockdown (Fig. 5e). Regarding the source of calcium ions, our data showed that releasing Ca^2+^ from ER by thapsagargin (TG) was unable to induce CKAP4 condensation (Supplementary Fig. S6g), and there were no evident nucleus-derived calcium signals under solid stress (Supplementary Fig. S6h, i). Thus, extracellular Ca^2+^ proved to be the major source of CKAP4 condensation.

Furthermore, to verify the interaction between calcium ions and CKAP4, the in silico analysis of charge was performed. As shown in Fig. 5f, there exists a number of negatively charged regions in CKAP4, and such negatively charged regions overlapped well with the cation binding sites (Fig. 5g, h). To prove this in cells, GFP-tagged CKAP4^FL^, and IDR-truncated variants were expressed in cells. Our Data showed that once IDR2, but not IDR1, was truncated, CKAP4 was unable to condense into speckles, suggesting a more important role of the IDR2 domain in stress responsiveness (Fig. 5i, j). To verify the effect of Ca^2+^ on CKAP4 condensation, direct interaction was studied on purified FL and IDRs-truncated constructs of CKAP4. As shown in Fig. 5k, the addition of Ca^2+^ considerably enhanced the condensation of CKAP4^FL^ and CKAP4^∆IDR1^, but not CKAP4^∆IDR2^. Altogether, these data revealed that CKAP4 condensation is highly associated with Ca_v_1.2-mediated calcium influx, and the charged pattern in IDR2 makes a significant contribution.

### CKAP4 condensation dramatically reorchestrates microtubule branching and lamellipodia formation

**Fig. 6:**
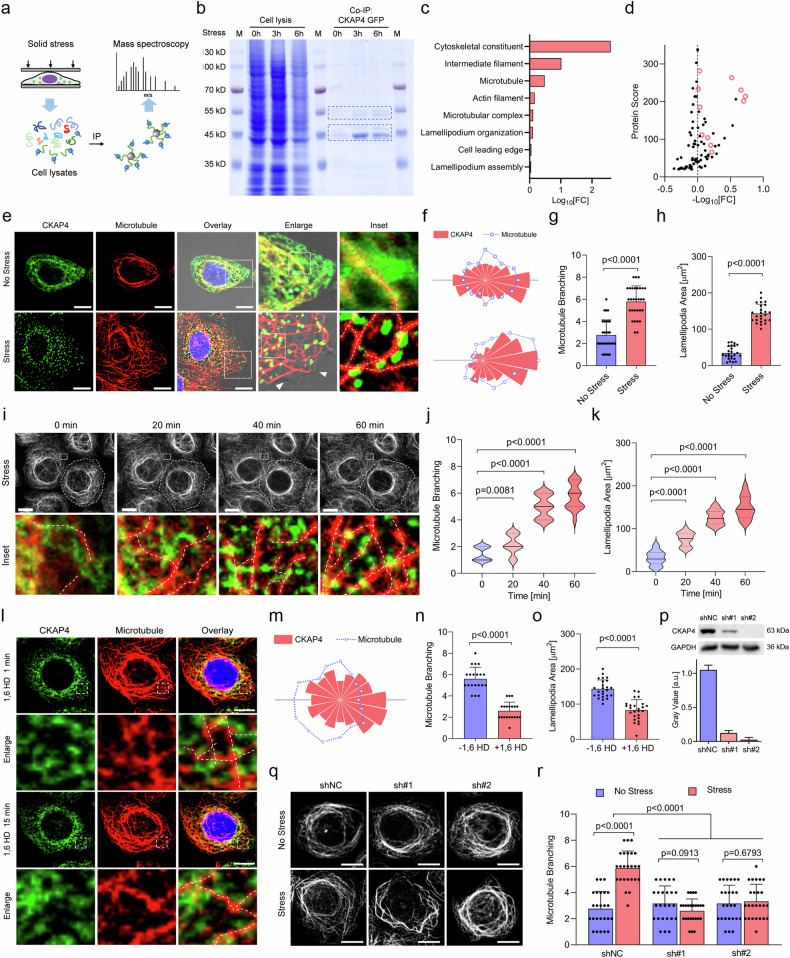
CKAP4 condensation dramatically reorchestrates microtubule branching and lamellipodia formation. **a** Method designed to identify the interacting proteins of CKAP4 under stress. **b** Coomassie blue-stained SDS-PAGE was used to analyze CKAP4-associated proteins. **c** GO analysis of interacting proteins in cells with or without stress. **d** Fold change of interacting proteins in cells with or without stress for 3 h. Cytoskeleton-associated proteins were marked as red circles. **e** Representative images of CKAP4-GFP-expressing 5637 cells with or without stress. Nuclei were stained with Hoechst, microtubules were shown in red. Scale bars, 10 μm. In the enlarged images, lamellipodia were indicated with white arrows. In the inset panel, microtubule branching was shown with a white dashed line. **f** Directional distribution of intracellular CKAP4 (red) and microtubules (blue) in (**e**) quantified by fluorescence intensity. **g** Microtubule branching analysis in cells with or without stress. More than 25 cells were calculated in each group. **h** Lamellipodia areas in cells with or without stress. More than 25 cells were calculated in each group. **i** Time-lapse imaging of CKAP4 condensation and microtubule branching in response to solid stress. The cell border was indicated with the dashed circle, and microtubules were shown in gray color. The selected area was enlarged in the bottom panel, where CKAP4 was shown in green, microtubules were shown in red, and branching was indicated with the dashed line. Scale bars, 10 μm. **j** Microtubule branching was calculated in a 3 × 4 μm window, within which the branching number was calculated. **k** Lamellipodia area was calculated according to the protrusion area on the cell border, more than 20 representative cells were included. **l** CKAP4 condensation and microtubule change in CKAP4-GFP expressing 5637 cells under stress after the treatment with 1,6 HD. Enlarged CKAP4-microtubule interaction and microtubule branching in the indicated areas were shown in the lower panel. Scale bars, 10 μm. **m** Directional distribution of intracellular CKAP4 and microtubules in cells treated with 1,6 HD in (**l**) quantified by fluorescence signals. **n** Microtubule branching analysis in cells under stress treated with or without 1,6 HD. **o** Lamellipodia areas in compacted cells treated with or without 1,6 HD. **p** Representative western blotting image of 5637 cells upon CKAP4 depletion. The bar chart was calculated by three independent experiments. NC, negative control. **q** Microtubule organization in 5637 NC or CKAP4 knockdown cells. Cells were treated with or without solid stress. Scale bars, 10 μm. **r** Microtubule branching analysis in the compacted cells upon CKAP4 depletion, and more than 20 representative cells were included. In **g**, **h**, **j**, **k**, **n**, **o**, **p**, **r**, data were represented as mean ± SD, and the *P*-values were calculated by Student’s *t*-test.

To uncover the biological significance of solid stress-induced CKAP4 phase separation, immunoprecipitation-mass spectroscopy (IP-MS) was used to identify interacted molecular components (Fig. 6a). Compared with CKAP4-associated proteins in unstressed cells, stressed cells showed enhanced enrichment of proteins at 45 kDa and 55 kDa (Fig. 6b). Gene ontology (GO) and molecular analysis showed that most changed proteins were cytoskeleton-associated proteins, including tubulin and actin (Fig. 6c, d). To unveil how CKAP4 condensates regulate cytoskeletons, microtubules and actin were fluorescently stained in CKAP4-GFP-expressing cells. As shown in Fig. 6e; Supplementary Fig. S7a, while both actin and microtubules were remodeled upon stress, microtubules showed more direct interactions with CKAP4 condensates, displayed as a spreading and branching pattern. Precisely, once CKAP4 condensed into speckles, CKAP4-microtubule colocalization was enhanced, and substantial lamellipodia were formed on the cell edge, while in unstressed cells microtubules showed a more curved and bundled state (Fig. 6e, h). Time-lapse imaging showed that microtubules were significantly branched out as the formation of CKAP4 condensation (Fig. 6i, k; Supplementary Fig. S7b–e). Once CKAP4 condensates were dissolved by 1,6-HD treatment, CKAP4-microtubule colocalization mitigated dramatically (Fig. 6l, m), and a great number of branched microtubules gradually returned to the bundled state together with reduced lamellipodia (Fig. 6n, o). These findings suggest the significance of CKAP4 condensates in microtubule organization. To further elucidate the causality between CKAP4 condensation and microtubule organization, both albendazole and colchicine were used to inhibit microtubule growth. Disruption of microtubules by either albendazole or colchicine could not block CKAP4 phase separation in stressed cells (Supplementary Fig. S7f, g), but knocking down of CKAP4 significantly disturbed the stressed cells to shift curved and bundled microtubules toward the spreading and branching pattern (Fig. 6p–r), which collectively proved that CKAP4 condensation is an upstream event able to regulate microtubule remodeling, not the other way round. Notably, when the applied solid stress was released by removing away coverslips, microtubule branching was gradually returned to the previous curved and bundled state together with the disruption of CKAP4 condensates (Supplementary Fig. S7h–j).

### CKAP4 condensation directly regulates microtubule branching in a size-dependent manner

**Fig. 7:**
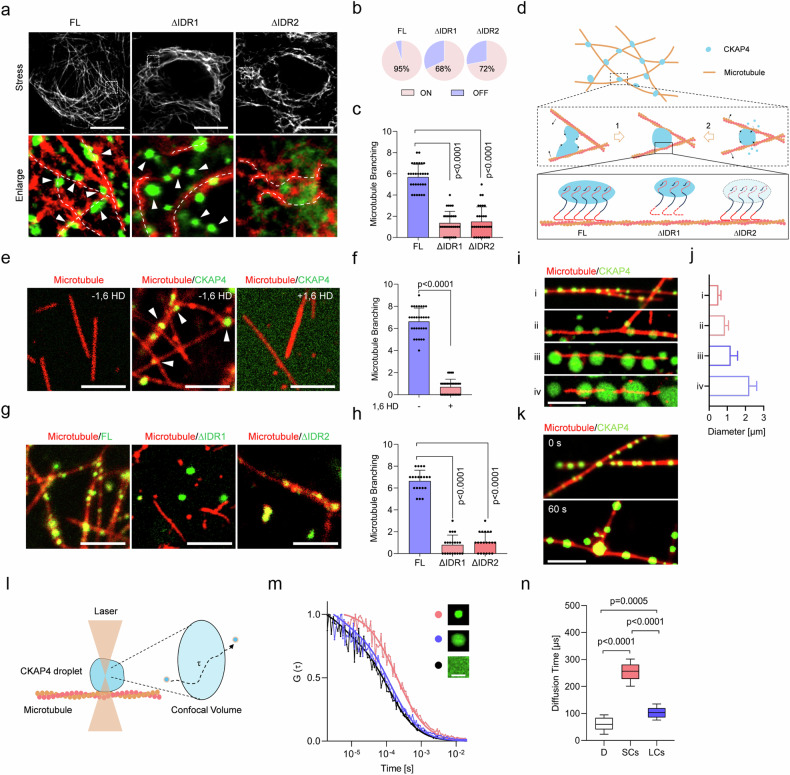
CKAP4 condensation directly regulates microtubule branching in a size-dependent manner. **a** CKAP4-depleted cells were re-expressed with GFP-fused CKAP4^FL^, CKAP4^∆IDR1^, and CKAP4^∆IDR2^. Microtubules were shown as gray color in the top panel, and interactions between the microtubule and CKAP4 condensates in the indicated region were shown at the bottom panel. The Dashed lines showed features of microtubules. White arrows indicated CKAP4 condensates. Scale bars, 10 μm. **b** Percentages of CKAP4 and its truncated variants on the microtubule. The pink color indicated CKAP4 ON the microtubule, while the blue color indicated CKAP4 OFF the microtubule. More than 20 cells were included for analysis. **c** Microtubule branching in CKAP4^FL^-, CKAP4^∆IDR1^-, and CKAP4^∆IDR2^-expressing cells, more than 20 representative cells were included in the quantification. **d** Schematic diagram of CKAP4 condensation in the modulation of microtubule branching. The middle panel represents two crucial molecular events illustrating how branching is regulated by interfacial tension. The bottom panel denotes the significance of the two IDRs in CKAP4-microtubule interactions, where IDR1 binds on microtubules, and IDR2 determines condensation capacity. **e** Effects of CKAP4 condensates on microtubule branching. 1% of 1,6 HD was used to disrupt CKAP4 condensation. Scale bars, 5 μm. **f** Microtubule branching number was calculated in the 10 × 10 μm area. **g** Effects of IDRs on microtubule branching. Scale bars, 10 μm. **h** Microtubule branching number was calculated in the 10 × 10 μm area. **i** Microtubule branching in the function of condensate size. CKAP4 condensates were shown in green, and microtubules were shown in red. Scale bars, 5 μm. **j** Condensate size was calculated in more than 30 condensates. **k** Time-lapse imaging of condensate in the modulation of microtubule branching. Scale bars, 5 μm. **l** schematic illustrating the use of FCS to measure molecular motions in condensates. **m** Molecular motile activity was measured by FCS in condensates of different sizes. **n** Diffusion time of molecules in condensates was calculated, and more than 20 condensates were included for calculation. D, diffusion, SCs, small condensates, LCs, large condensates. In **c**, **f**, **h**, **n**, data were represented as mean ± SD, and the *P*-values were calculated by Student’s *t*-test.

To investigate the modulation of microtubules by CKAP4 condensates, endogenous CKAP4 was depleted, and then CKAP4^FL^ and its truncated variants were re-expressed in cells, respectively. As shown in Fig. 7a, it was CKAP4^FL^, rather than its variants (CKAP4^∆IDR1^ or CKAP4^∆IDR2^), that could regulate microtubule branching. Particularly, although CKAP4^∆IDR1^ retained the ability to form condensates, its interaction with microtubules was disrupted, making it impossible to reorganize microtubules; in the case of CKAP4^∆IDR2^, it was not even able to form condensates (Fig. 7b, c). Therefore, the regulation of microtubules requires IDR2 for phase separation and IDR1 for binding on microtubules. Then, once condensation is initiated on microtubules, interfacial tension-based CKAP4 mechanical effectors can be generated for microtubule spreading and branching, as illustrated in Fig. 7d. To further prove this assumption, an in vitro test with tubulin and CKAP4 constructs was performed. Firstly, microtubules were produced in advance, once CKAP4 condensates were added, they attached to microtubules and produced branched microtubules. However, neither condensate-free CKAP4 solution nor 1,6 HD-treated CKAP4 could induce microtubule branching (Fig. 7e, f). Consistent with the observation in cells, it was CKAP4^FL^, rather than its IDR-truncated variants, that could regulate microtubule branching (Fig. 7g, h).

We also noticed that tiny CKAP4 condensates (< 1 μm) were mainly involved in the regulation of microtubule branching. Therefore, we asked whether these tiny droplets would exhibit more formidable effects on microtubule reorganization. To address this question, obtained microtubules were respectively added into CKAP4 condensates of various sizes, and only the tiny speckles (< 1 μm) showed sufficient ability to induce microtubule branching, whereas those larger than 1 μm could only able to attach on microtubules, but failed to induce microtubule branching (Fig. 7i, j). Time-lapse imaging showed that as CKAP4 condensates grew, contracted, collided, or fused, they, especially those at intersection points, could tune the degree between two microtubules through interfacial tension (Fig. 7k). To further investigate size effects on the physical features of condensates, molecular diffusion in the condensates was examined by fluorescence correlation spectroscopy (FCS) in vitro. Our data showed that molecules in small condensates were inactive, with a diffusion time (253.7 ± 33.7 μs) significantly slower than that of large condensates (103.2 ± 19.4 μs) and that in solution (66.6 ± 25.5 μs), as shown in Fig. 7l–n. This means that intermolecular interaction in small condensates conferred sufficient stability to endow these condensates with enough mechanical strength to remodel microtubules.

### Solid stress enhances cancer metastasis by CKAP4 phase separation

**Fig. 8:**
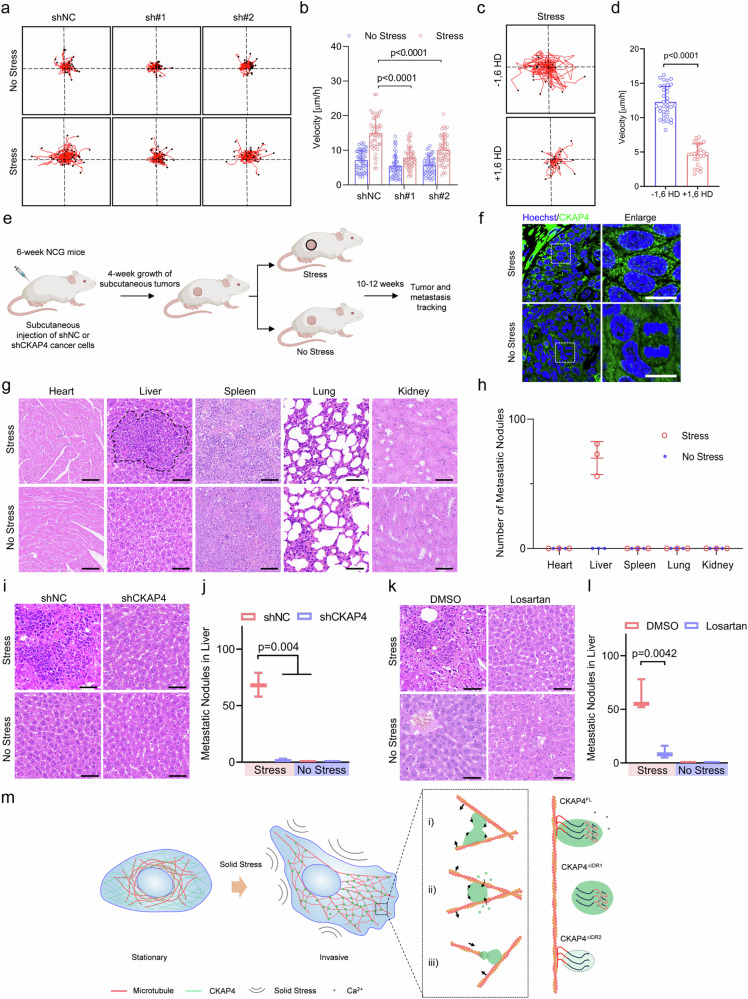
CKAP4 condensation mediates solid stress-driven cancer metastasis. **a** CKAP4 mediates single-cell migration of 5637 cells under stress. More than 20 individual cells were tracked for 12 h under microscopy. **b** Migration velocity analysis of 5637 cells in (**a**). **c** Single-cell migration of 5637 cells under stress pretreated with or without 1,6 HD for 1 min. More than 20 individual cells were tracked for 12 h under microscopy. **d** Migration velocity analysis of 5637 cells in (**c**). **e** Schematic and schedule of in vivo study. **f** Representative images of CKAP4 condensation in tumors with or without solid stress. Scale bars, 10 μm. **g** The metastasis of cancer cells in the indicated organs was determined by H&E staining. Representative images of three independent experiments are shown. Scale bars, 50 μm. **h** Quantification of metastatic nodules in (**g**). *n* = 3. **i** Representative images of metastatic nodules of shNC or sh*CKAP4* 5637 cancer cells in the liver determined by H&E staining. Scale bars, 50 μm. **j** Quantification of metastatic nodules in (**i**). **k** Representative images of metastatic nodules of 5637 cancer cells in the liver determined by H&E staining. Losartan was intravenously injected to release the solid stress in tumor sites. Scale bars, 50 μm. **l** quantification of metastatic nodules in (**k**). Data were represented as mean ± SD, and the *P*-value was calculated by Student’s *t*-test. **m** Model of CKAP4 condensation in mediating microtubule organization and driving cancer metastasis. In **b**, **d**, **h**, **j**, **l**, data were represented as mean ± SD and the *P*-values were calculated by Student’s *t*-test.

Based on the above-mentioned observations, cancer cells under solid stress formed a considerable number of lamellipodia on the cell edge, which was in line with the significant association of CKAP4 and cell migration in GO analysis. Thus, these data provided clues on CKAP4-mediated metastasis as shown in Fig. 1. To elucidate the involvement of CKAP4 in solid stress-promoted cancer metastasis, single-cell motility was investigated. Fig. 8a, b showed that CKAP4 depletion significantly mitigated cell motility in both stressed and unstressed cells; particularly, in the stressed group, the motile velocity of CKAP4-depleted cells was almost half that of the NC group, with a similar level of unstressed shNC cells. This could be attributed to the loss-of-function of mechanical architecture in stressed cells by CKAP4 depletion. Likewise, when CKAP4 phase separation was inhibited by 1,6 HD, the mobility of stressed cells decreased almost 3 times compared with that of untreated cells, further emphasizing the major influence of CKAP4 condensation on cell motility. In parallel, in the wound healing assay and transwell assay, stressed cells showed more significantly enhanced migration than that of unstressed cells (Supplementary Fig. S8a–e). Interestingly, as shown in the inset image of cells located in the pores of the transwell, CKAP4 was mainly exhibited as condensates (Supplementary Fig. S8d).

To investigate whether CKAP4 phase separation-mediated microtubule branching promotes cell migration in 3D culture, spheroids were generated by CKAP4-GFP expressing 5637 cells. As shown in Supplementary Fig. S8f–h, in sparsely-compacted spheroids, CKAP4 did not undergo phase separation, but in highly-compacted spheroids with bigger sizes there showed obvious CKAP4 condensates, and in phase separation, there was a greater proportion of stretched cells, which potentially pointed out the significance of microtubule branching. To further verify this, spheroids were embedded in agarose-based hydrogel. When spheroids were stressed by hydrogel, CKAP4 underwent phase separation even in a smaller size compared with those suspended in cell media. In line with the findings in 2D culture, CKAP4 condensates promoted microtubule branching even in 3D culture, and as a result, there was a remarkable number of cells moving onto the surface of spheroids for migration; by contrast, when microtubules were disrupted by colchicine, stretched cells, and migration sites onto the surface of spheroids were reduced sharply (Supplementary Fig. S8i–k). Therefore, CKAP4 phase separation-mediated microtubule branching was proved to be one crucial driver for cell migration, and eventually metastasis.

To further verify the role of CKAP4 phase separation and solid stress in cancer metastasis, we designed and performed the experiment in vivo, as shown in Fig. 8e; Supplementary Fig. S9a. Indeed, in stressed tumor CKAP4 showed evident phase separation (Fig. 8f), and it led to a dramatic distant metastasis to the liver (Fig. 8g, h), CKAP4 exhibited a significant effect (Fig. 8i, j; Supplementary Fig. S9b, c). By contrast, when mice were treated with losartan, one of the FDA-approved antihypertensive drugs that can release the solid stress in tumors, cancer metastasis was significantly alleviated (Fig. 8k, l; Supplementary Fig. S9d, e). Altogether, our data suggested that solid stress-promoted cancer metastasis by inducing microtubule branching, during which CKAP4 condensation played an important role (Fig. 8m).

## Discussion

In this study, we identify a crucial mechanical sensor, termed CKAP4, underpinning solid stress-induced cancer malignancy. Different from previously identified surface sensors (e.g., Integrins, Piezos, and FAKs), CKAP4 is surprisingly intracellularly located. This is an interesting finding, as it refreshes the conventional knowledge of cell membrane-localized mechanical sensors, and illustrates a paradigm of how cancer cells sense solid stress and activates downstream signaling.

Furthermore, CKAP4 condensation shows strong specificity to solid stress among a subset of studied TME characteristics in this work. Surprisingly, none of the other broadly investigated physical stimuli, such as cell packing and substrate stiffness, are able to initiate phase separation. This is different from some elsewhere reported mechanically associated condensates, e.g., YAP and TAZ, which can also be triggered by serum starvation, substrate stiffness, and cell packing^18^. Our experimental evidence helps to discriminate some seemingly similar physical triggers in tumors, namely solid stress, cell packing, substrate stiffness, and space confinement. Importantly, as a mechanical sensor, CKAP4 is more sensitive than the nucleus-based mechanical ruler (2 nN for CKAP4 and 100 nN for nucleus)^40^. CKAP4 is potentially to be a robust molecular identification to study solid stress-associated cancer biology.

Based on our observations, this mechanosensing mechanism depends on a three-dimensional stress on cells, rather than side-by-side stress derived from increased cell density or adhesion/traction forces from substrate stiffness. As a result, cell height is reduced under stress, and intracellular calcium ions are dramatically accumulated. Particularly, it is extracellular Ca^2+^, rather than the ER- or nucleus-derived Ca^2+^, that fluxes into cytosol and plays dominant roles in driving CKAP4 condensation. Since CKAP4 condensation can be initiated by the agonists of the Ca_V_ family, but not those of the Piezo and TRP family, either by L-type calcium channel inhibitors (isradipine or azelnidipine), or Ca_v_1.2 knockdown, CKAP4 condensation was significantly inhibited, suggesting the critical role of Ca_v_1.2 in solid stress-associated cancer biology^41^. Apart from the identified role of Ca_v_1.2 in some mechanical contexts as a key regulator of heart rate, rhythm, and force of contraction, we further uncover its novel function responding to solid stress in cancer. In this study, we also prove that the negatively charged IDR2 domain of CKAP4 interacts strongly with Ca^2+^, and makes the major contribution to initiate condensation, which is in line with the patterned charge blocks and calcium transients for phase separation in some other studies^42–44^. And the coiled IDR2 region is folded into a cluster enriched in negative charge that benefits robust IDR2–Ca^2+^ interaction. Altogether, the calcium ion-dependent CKAP4 phase separation denotes a special mechanism for cancer cells to sense solid stress from within cells, which proves to be an important missing block in cancer biomechanics.

Another notable finding is the identification of microtubule branching in response to solid stress. As far as our knowledge, the role of microtubule branching in driving cancer metastasis, especially in the context of solid stress, remains elusive. Our study uncovers that it is LLPS-regulated microtubule branching, rather than previously reported microtubule nucleation^40,45^, growth^25^ or stability^26,46^, that constitutes one of the major intracellular traits decoding solid stress-associated cancer cell biology. CKAP4 condensates adhere firmly onto microtubules and become the hubs that shift curved and bundled microtubules into the straight and branching pattern through interfacial tension. As a result, cancer cells are maintained in a stretched state in both 2D and 3D cultures. In this way, cells are endowed with a stronger ability to sense the external environment of less stressed regions due to the increased cell spreading area. Following this clue, future studies should exert more effort on the spatiotemporal organization of microtubule-associated condensates, condensate physics, and solid stress-induced cancer progression. Interestingly, we also notice that cancer cells tend to use a flattened, lamellipodial mode for migration under solid stress. Once such stress is released or migrated to the surface of spheroids, cells appear to shift into a blebbing-based mode. Our study offers additional insights into the features of cancer cell migration, particularly in the context of solid stress, which is so far poorly understood.

Regarding CKAP4, previous studies have reported it as a molecular receptor of APF, DKK1, and etc. in signaling pathway^31^, and it also shapes ER sheet^28^ and organizes organelle-to-organelle interactions^34,35^. Apart from this knowledge, our study discovers its novel role in mechanical sensing by forming condensates, which opens up new directions for the study of this oncoprotein in driving cancer metastasis and further diversifies the molecular family of mechanobiology, particularly an intracellular mechanosensor. Moreover, instead of PTMs or activating downstream molecular signaling, CKAP4 condensates per se can directly organize microtubule remodeling. Attached to microtubules, submicron-leveled CKAP4 condensates exhibit biophysical ability to influence microtubule bundles along with their growth, collision, fusion, and fission. Our data also show that small condensates are more potent to branch microtubules than the large ones, which further emphasizes the role of physical traits of CKAP4 condensates, e.g., interfacial tension and viscoelasticity, in mechanical regulation^47–50^. However, due to the small size and technical challenges, a quantitative comparison is lacking in our study.

In cancer mechanobiology, to date, direct in situ measurement of solid stress in tumor tissues is still challenging. Considering the specific response of CKAP4 condensation, it can be used as a mechanical indicator to map the mechanical landscape of solid stress in tumors. Compared with the nucleus-based organelle leveled mechanical ruler^40^ or cell membrane-localized biosensor, e.g., Piezos^51^, the CKAP4-based mechanical ruler provides an alternative approach for intracellular biological applications.

In summary, our study bridges the gap between solid stress and cancer metastasis by CKAP4 phase separation-mediated microtubule branching, and the discovery of a CKAP4-based intracellular sensor for solid stress in tumors provides a new insight into the biomechanics to drive cancer progression. Our study not only diversifies the protein family of mechanobiology but also identifies a potential treatment target to meliorate clinical outcomes. Deciphering the biomolecular codes underpinning cancer physics opens up new avenues for clinical treatment, particularly in the context where solid stress has been proven of great significance in malignant progression^3,11^.

## Materials and methods

**Key resources table**

**Table:** 

Antibodies	Sources	Identifier
Anti-CKAP4 (For WB) Rabbit	Sangon Biotech	Cat# D261564
Anti-CKAP4 (For IF) Rabbit	Proteintech Group	Cat# 16686-1-AP
Anti-Phosphoserine Mouse	Santa Cruz Biotechnology	Cat# sc-81514

Chemicals	Sources	Identifier
RPMI-1640	Gibco	Cat# 11875176
Dulbecco’s Modified Eagle’s medium (DMEM)	Gibco	Cat# 11330032
Fetal Bovine Serum (FBS)	Gibco (U.S. origin)	Cat# 900-108, 90010
Penicillin-Streptomycin(P/S)	ThermoFisher Scientific	Cat# 15140163
D-PBS (Free-Ca^2+^/Mg^2+^)	ThermoFisher Scientific	Cat# 14190144
Lipofectamine RNAiMAX	ThermoFisher Scientific	Cat# 13778030
0.25%Trypsin-EDTA	ThermoFisher Scientific	Cat# 25200114
Protease Inhibitor Cocktail	Cell signaling	Cat# 5871
RIPA Lysis and Extraction Buffer	ThermoFisher Scientific	Cat# 89900
Phosphatase Inhibitor (EDTA-Free)	Bimake	Cat# B14001
Hoechst	Beyotime Biotechnology	Cat# C1011
GTP	Beyotime	Cat# 36051-31-7
PEG 5000	Merck	Cat# 198227-38-2
1,6-Hexanediol	Sigma	Cat# 8043080100
BAPTA-AM	ThermoFisher Scientific	Cat# B1205
Yoda1	MedChemExpress	Cat# 448947-81-7
PF-573228	MedChemExpress	Cat# 869288-64-2
Tubulin Tracker^®^ Deep Red	Invitrogen	Cat# T34077
SiR-Actin Kit	Cytoskeleton	Cat# CY-SC001
Tubulin (HiLyte 647 dye labeled; porcine)	Cytoskeleton	Cat# TL670M-A
ER-Tracker Red	ThermoFisher Scientific	Cat# E34250
Cal-590	AAT Bioquest	Cat# 20510
Thapsagargin (TG)	Cell Signaling Technology	Cat# 12758S
Colchicine	MedChemExpress	Cat# HY-16569
Capsaicin	MedChemExpress	Cat# HY-10448
GSK1016790A	MedChemExpress	Cat# HY-19608
Curcumin	MedChemExpress	Cat# HY- N0005
GV-58	MedChemExpress	Cat# HY-12498
Urolithin C	MedChemExpress	Cat# HY-135897
(S)-(-)-Bay-K-8644	MedChemExpress	Cat# HY-15124

Recombinant proteins	Sources	Identifier
GFP	This Research	N/A
CKAP4-FL	This Research	N/A
CKAP4-∆IDR1	This Research	N/A
CKAP4-∆IDR2	This Research	N/A

Cell lines	Sources	Identifier
SV-HUC-1	CAS Cell Bank	N/A
5637	CAS Cell Bank	N/A
T24	CAS Cell Bank	N/A
TCC-SUP	ATCC	N/A
TPC-1	ATCC	N/A
MCF-7	ATCC	N/A
U2OS	ATCC	N/A
A549	ATCC	N/A
Hela	ATCC	N/A
K562	ATCC	N/A
HEL	ATCC	N/A
Ramos	ATCC	N/A
CEM	ATCC	N/A
trelief ™ 5α chemically competent cell	Tsingke Biotechnology	N/A
*E. coli Rosetta* 2(DE3)	Tsingke Biotechnology	N/A

Recombinant DNA/Plasmid	Sources	Identifier
CKAP4-GFP	This Research	N/A
GFP-CKAP4	This Research	N/A
pCDH-CMV-MCS-EF1-Puro	YouBio	N/A
pCDH-CMV-MCS-EF1-Puro-CKAP4-FL	This Research	N/A
pCDH-CMV-MCS-EF1-Puro-CKAP4-∆IDR1	This Research	N/A
pCDH-CMV-MCS-EF1-Puro-CKAP4-∆IDR2	This Research	N/A
pET28a	This Research	N/A
pET28a-CKAP4-FL	This Research	N/A
pET28a-CKAP4-∆IDR1	This Research	N/A
pET28a-CKAP4-∆IDR2	This Research	N/A

Research and Assay Kit	Sources	Identifier
inNova UScript II First-Strand cDNA Synthesis SuperMix (with gDNA Removal)	Hunan Innovagene AB Biotechnology Limited	Cat# AR121-Mix
ClonExpress® II One Step Cloning Kit	Vazyme Biotech	N/A
Ni-NTA Sefinose^TM^ Resin	Sangon Biotech	Cat# C600332

Software and algorithms	Sources	Identifier
ImageJ	NIH	https://imagej.nih.gov/ij/
GraphPad Prism Software	GraphPad	https://www.graphpad.com/scientificsoftware/prism/
PyMOL	PyMOL	http://pymol.sourceforge.net/

### Ethics statement

The Ethics Committee of Central South University approved this study (2022-2-9 and 2022-1-5). All experiments performed in this study conform to related ethical guidelines. The maximal tumor size of animal experiments was 2000 mm^3^. The paraffin-embedded cancer tissue samples mentioned were obtained from the Pathology Department of Xiangya Hospital.

### Cell culture

5637, T24, TCC-SUP, PC-3, A549, U2OS, CEM, K562, HEL, Ramos, and TPC-1 cells were cultured in Roswell Park Memorial Institute (RPMI-1640) medium supplemented with 10% FBS. MCF-7 cells and HeLa cells were cultured in DMEM medium supplemented with 10% FBS. SV-HUC-1 cells were cultured in MEM medium supplemented with 10% FBS. Cells were plated either in 6-well, 24-well, 96-well tissue culture plates or on glass coverslips, and they were incubated at 37 °C with 95% humidity and 5% CO_2_.

### Plasmid construction

DNA fragments encoding human CKAP4 were PCR-amplified from 5637 cell cDNA using inNova UScript II First-Strand cDNA Synthesis SuperMix (with gDNA removal). The CKAP4-FL fragment was PCR-amplified and the DNA fragments encoding CKAP4 were inserted into the pCDH-CMV-MCS-EF1-Puro backbone by using the ClonExpress® II One Step Cloning Kit. Truncated mutants of pCDH-CMV-MCS-EF1-Puro-CKAP4-∆IDR1, and pCDH-CMV-MCS-EF1-Puro-CKAP4-∆IDR2 were created from pCDH-CMV-MCS-EF1-Puro-CKAP4-FL by overlapping extension-PCR methodology. The plasmid construction of the prokaryotic expression system was similar to that constructed above, but the vector was changed to pET28a.

### Cell line construction

CKAP4-GFP and mutants overexpressing 5637 cell lines were generated by plasmids transfection. Puromycin was used to screen the transfected cells with the gene of interest, according to the manufacturer’s protocol. Flow cytometry-assisted cell sorting was used to enrich CKAP4-GFP expressed cells.

Endogenous CKAP4 expression was knocked down by using a lentiviral packaging shRNA expression vector (purchased from GenePharma) to transduce the cells. 1.5 × 10^5^ target cells were seeded in wells of a 6-well cell culture plate and infected with lentivirus for 72 h. The following shRNA target sequences were used: *CKAP4*#1, 5′-CCAAATCCATCAACGACAA-3′; *CKAP4*#2, 5′-AACTTTTGAGTCCATCTTGAGAA-3′; and negative control sequence, 5′-TTCTCCGAACGTGTCACGT-3′. After 3 days, cells were selected by puromycin (20 μg/mL) to obtain stable knockdown cells.

### Bacterial cell culture

cDNA clones were transformed into *E. coli* (trelief™ 5α chemically competent cell). Single colonies were grown overnight at 37 °C in LB media containing selection antibiotic (50 mg/mL ampicillin for pCDH-CMV-MCS-EF1-Puro and 50 mg/mL kanamycin for pET28a). Cells were pelleted by centrifugation and lysed for harvesting DNA. All cells were stored at −80 °C until transformation for cloning and recombinant protein for expression.

### Protein purification

CKAP4-FL and mutants were expressed and purified from *E. coli* Rosetta 2 (DE3) cells and purified under native conditions unless otherwise noted. The constructs of CKAP4-FL and mutants contain a TEV cleavage site between the C-terminal 6× His and fusion protein and were grown to OD_600_ of 0.8 and induced with 0.6 mM IPTG at 16 °C or 4 °C overnight. Pelleted cells were resuspended in lysis buffer (protease inhibitor). After sonication, lysates were pelleted at 30,000× *g* at 4 °C for 30 min. Supernatants were purified following the Ni-NTA Sefinose^TM^ Resin protocol. The fractions were analyzed by SDS-PAGE, pooled, and concentrated. The fractions were stored at −80 °C.

### Solid stress by AFM

To study the solid stress, force magnitude, and stress elasticity were measured by AFM.

To investigate CKAP4 condensation of cells in response to external force, a tipless MLCT-10 probe (Bruker, Germany) was used to stress cells. Briefly, the probe was first calibrated by thermal noise signals. Then, a series of applied forces (from 0.1 to 20 nN) by AFM probes on cells were set, and time-lapse imaging of CKAP4 condensation was performed with an Olympus confocal microscope. In our study, stress mode, i.e., continuous stress and intermittent stress (with a time interval of 30 s) was investigated, respectively. More than 10 representative cells were examined.

AGs of different concentrations (0.5%, 1%, 2%, and 5%) and glass were prepared by dissolving agarose powder in water. Then, a layer of hot agarose solution was added to the culture dish to get a proper coating of AG after cooling down. Then, the elasticity of AGs was measured by AFM. The force spectra were mapped with a DNP-10 probe (Bruker, Germany) by the JPK Nanowizard 4 atomic force spectroscope. Before measuring Young’s modulus, the spring constant of the cantilever was first calibrated by thermal noise signals. In this scenario, force curves were collected on the Petri dish filled with ultrapure water. Afterward, AG-coated dishes were washed and refreshed with fresh PBS. The setpoint was 2.0 nN, *Z* length was 2000 nm, and *Z* speed was 60 μm/s for measurement. The scan area was 10 × 10 μm with a pixel of 32. To interpret the data, the Hertz model was chosen for fitting the force curve with the JPKSPM data processing software.

### In silico analysis of CKAP4 condensation

LCD was analyzed by SMART (http://smart.embl.de/). Scores for the predicted disorder plotted in Figs. 3j and 4d were obtained using Predictor of Natural Disordered Regions (PONDR) (http://www.pondr.com). Charge plots in Fig. 4d were prepared using the EMBOSS Charge tool (https://www.bioinformatics.nl/cgi-bin/emboss/charge) with a window size of 20. CKAP4-metal ion interaction was analyzed by the online program BioMetAll^52^. The structure of CKAP4 was simulated by AlphaFold2.

### Calcium channel treatment

To investigate the role of extracellular calcium ions in initiating CKAP4 phase separation, calcium inhibitors, i.e., isradipine and azelnidipine, were used to inhibit the calcium influx. Briefly, GFP-CKAP4-expressing 5637 cells were seeded onto the bottom of the culture dish overnight for attachment. Afterward, cells were treated with calcium channel inhibitors, isradipine, and azelnidipine, of the indicated concentrations, or DMSO for control, for 2 h. Then, cells were stressed with glass coverslips for 1 h. CKAP4 phase separation was observed under a confocal microscope.

To verify the dominant calcium channels mediating CKAP4 phase separation, various available calcium channel agonists were used for cell treatment. Briefly, GFP-CKAP4-expressing 5637 cells were seeded onto the bottom of the culture dish overnight for attachment. Afterward, cells were treated with calcium channel agonists, Yoda1, Capsaicin, GSK1016790A, Curcumin, GV-58, Urolithin C, (S)-(-)-Bay-K-8644, of the indicated concentrations, or DMSO for control, for 1 h. Then, CKAP4 phase separation was observed under a confocal microscope.

To further identify the role of L-type calcium channel Ca_v_1.2 in CKAP4 phase separation, siRNAs were used to knockdown the level of Cav1.2 in GFP-CKAP4-expressing 5637 cells (Ca_v_1.2_1, CTGGTTTGGTTCGGTTATCTAdTdT; Ca_v_1.2_2, TCCAGGGATGTTAGTCTGTATdTdT)^53^.

To investigate the role of ER calcium ions in CKAP4 phase separation, TG was used to increase cytosolic Ca^2+^ concentrations via the discharge of intracellular Ca^2+^ stores^54^. Briefly, cells were firstly treated with calcium-depleted medium for 1 h, then they were treated with TG of indicated concentration for as long as 1 h. Intracellular CKAP4-GFP was tracked under a microscope.

### ICP-OES

2 × 10^6^ cells in 2 mL cell media were seeded in 6-well plates overnight for attachment. Then, cells were treated with solid stress or Yoda1 at the indicated times or doses. To measure the intracellular concentration of Ca^2+^, cells were firstly washed 3 times with 2 mL of MilliQ water to remove all Ca^2+^-containing medium. Afterward, 100 μL of 1% HCl solution was used to get the cell lysates. Collect all the suspension, and dilute it with MilliQ water to make a final volume of 3 mL. After a brief centrifugation to remove cell debris, calcium concentration was measured by ICP-OES.

### CKAP4 droplet formation in vitro

CKAP4^FL^, CKAP4^∆IDR1^, CKAP4^∆IDR2^, and CKAP4^∆IDR1/2^ solution of the indicated protein concentrations were prepared with the Tris-HCl buffer (pH 7) containing PEG 5000, respectively. After 10 min incubation at room temperature, 2 μL of solution was added to the bottom of the ibidi imaging dish. Droplet formation was observed under a confocal microscope with a 60× objective lens. As a negative control, the condensation behavior of CKAP4 was investigated in the inhibiting solution (1% of 1,6 HD, 20% of PEG 5000, Tris-HCl buffer, pH 7). To investigate the effect of calcium ions on droplet formation, CaCl_2_ in Tris-HCl buffer containing 20% PEG 5000 at the indicated concentrations were prepared, respectively. To investigate the fusion and growth of CKAP4 droplets, time-lapse imaging was recorded for 10 min with a time interval of 5 s. Images were analyzed with ImageJ.

### CKAP4 condensation in tumor tissues from BLCA and LUAD patients

To investigate solid stress and CKAP4 condensation in tumor tissues of cancer patients, tumor sections of BLCA and LUAD were generated using a vibratome slicing system. After deparaffinization with various concentrations of ethanol, sections were stained with H&E, Hoechst, and the CKAP4 antibody. Stress level and CKAP4 condensation were observed under a confocal microscope, images were analyzed with ImageJ.

### In vitro test of CKAP4 condensates on microtubule organization

Fluorescently labeled microtubules were produced based on the manufacturer’s instructions. Briefly, HiLyte Fluor^TM^ 647 labeled tubulin was resuspended in 5 µL of General Tubulin Buffer plus GTP (G-PEM) plus 10% glycerol buffer on ice. Then, the solution was placed at 37 °C for 20 min. Afterward, 0.7 µL of 200 µM taxol in G-PEM plus 10% glycerol solution was added and incubated at 37 °C for 5 min. This is the microtubule stock solution. Then, 1 µL of MT stock solution was diluted into 200 µL of 37 °C warm G-PEM buffer plus 30% (w/v) glycerol and 20 µM taxol to generate 5–10 µM long microtubules.

To investigate the effect of CKAP4 condensates on microtubule organization, 5 µL of as-prepared microtubules was added into 5 µL of CKAP4 condensates suspension (80 µg/mL of CKAP4-FL or truncated variants, 20% PEG 5000, Tris-HCl buffer, pH 7). Since proteins were not labeled with GFP, a CKAP4-specific aptamer FITC-spl3c (final concentration 0.5 µM) was included in the buffer for fluorescence tracking. After 5 min incubation at room temperature, 5 µL of solution was added to the bottom of the ibidi imaging dish for microscopic observation. Images were analyzed by ImageJ.

### FCS

FCS measurement was used to quantify the dynamics and concentration of CKAP4 in condensates, which was performed under a 40× water-immersed objective lens with Zeiss LSM 880. Before use, parameters, such as pinhole size, confocal volume, and standard curves, were calibrated with an FITC solution. To measure the dynamics of CKAP4, condensates of various sizes were examined. For each FCS measurement, 10% laser intensity was used at the objective outlet, and samples were exposed to the laser for 30 s to obtain the autocorrelation curve, during which a stable fluorescence signal was monitored to avoid photobleaching. For each position, 3 individual curves were collected and averaged. More than 20 condensates were included in each group.

1 $$G(\tau )=\frac{1}{N}\left(\frac{1}{1+\frac{\tau }{{\tau }_{D}}}\right)\left(\frac{1}{\sqrt{1+\frac{{\omega }^{2}\tau }{{z}_{0}^{2}{\tau }_{D}}}}\right)$$

The term τ_D_ is the characteristic correlation time during which a molecule resides in the observation volume of radius ω and length *z*_0_, as given by τ_D_ = ω^2^/4*D*, where *D* is the diffusion coefficient and *N* is the mean number of fluorescent particles in the confocal volume. When τ → 0, *G* (0) is yielded.

2 $$G(0)=\frac{1}{N}$$

Therefore, based on the fitting curve of the autocorrelated spectra, the mean number of FITC-aptamers in the confocal volume can be obtained. As a result, the density of CKAP4 in condensates can be estimated.

To measure the concentration of CKAP4 in condensates, a standard curve was first made as a function of FITC-aptamer concentrations.

### FRAP

FRAP experiments were performed for cells expressing wild-type or mutant GFP-CKAP4. Time series data for FRAP experiments were acquired using 20 cycles of 1 min intervals, during which the eGFP signal was bleached using a 488 nm laser with 100% intensity after the second interval. Fluorescence intensities were acquired from around ten regions of interest, quantified using ZEN Black software and reported as relative values to the pre-bleaching time point.

### Immunofluorescence

For Immunofluorescence, cells cultured on glass coverslips were fixed with 4% PFA (P0099, 4% PFA Fixing Solution, Beyotime Biotechnology) for 15 min. Cells were then washed three times with D-PBS (Ca^2+^/Mg^2+^ free) for 5 min and permeabilized with 0.1% Triton X-100 at room temperature for 5 min. Cells were then washed three times with D-PBS (Ca^2+^/Mg^2+^ free) and then blocked with 1% bovine serum albumin at room temperature for 1 h. Primary antibodies against CKAP4 (16686-1-AP, 1:100 dilution, Proteintech) were incubated at 4 °C for 8 h. The binding of primary antibodies was visualized via Alexa Fluor 488 conjugated secondary IgG antibodies with 1 h incubation at room temperature. Nuclei were then stained with Hoechst (4 μg/mL, 33258, Beyotime Biotechnology) at 37 °C for 15 min.

### Western blot analysis

For immunoblotting, cells were seeded in wells of a 6- or 12-well plate. Protein samples were collected using the RIPA lysis buffer with protease inhibitor cocktail (1:20 dilution). Samples were centrifuged at 4 °C for 15 min and the protein content quantification was performed using Nanodrop (ThermoFisher Scientific). Proteins were subjected to SDS-PAGE electrophoresis (10%, Bio-Rad) and transferred to a PVDF membrane. Immunoprobing was performed using rabbit polyclonal CKAP4 antibody (1:2000), mouse polyclonal GAPDH (1:5000), mouse polyclonal p-serine antibody (1:200), and mouse polyclonal GFP (1:200), followed by binding with horseradish peroxidase-conjugated anti-rabbit antibody and anti-mouse antibody (1:1000). Films were detected with the ECL substrate kit and ChemiDoc XRS+ System (1708265, Bio-Rad).

### IP-MS

After washing 2 times with cold D-PBS, 5637 cells with or without stress were dissociated with 0.2% EDTA and lysed in 5 mL of hypotonic buffer (D-PBS 50 mM, Tris-HCl (pH 7.5)) containing 0.1 mM PMSF at 4 °C for 30 min. After centrifugation, the debris was washed three times with 5 mL of hypotonic buffer and dissolved in 1.5 mL of lysis buffer (D-PBS containing 5 mM MgCl_2_ and 1% Triton X-100) at 4 °C for 30 min. After collection by centrifugation, the proteins were eluted by heating in 30 μL of loading buffer and analyzed by SDS-PAGE gel and Coomassie Blue staining. The bands of interest were excised for digestion in situ and analyzed by LC-MS/MS.

The lyophilized peptide fractions were resuspended in ddH_2_O containing 0.1% formic acid, and 2 μL of which was loaded into a nanoViper C18 (Acclaim PepMap 100, 75 μm × 2 cm) trap column. The online Chromatography separation was performed on the Easy nLC 1200 system. The trapping, desalting procedure were carried out with a volume of 20 μL 0.1% formic acid. Then, an elution gradient of 5%–38% in a solvent containing 80% acetonitrile and 0.1% formic acid in 60 min was used on an analytical column. DDA (data-dependent acquisition) mass spectrum techniques were used to acquire tandem MS data on a ThermoFisher Q Exactive mass spectrometer fitted with a Nano Flex ion source. Data were acquired using an ion spray voltage of 1.9 kV, and an interface heater temperature of 275 °C. For a full survey scan, target value was 3 × 10^6^, ranged from 350 to 2000 m/z at a resolution of 70,000 and a maximum injection time of 100 ms. The MS2 spectra were acquired in the ion trap in rapid mode with an AGC target of 8000 and a maximum injection time of 50 ms. Dynamic exclusion was set for 25 s. The local false discovery rate at PSM was 1.0% after searching against Homo sapiens database with a maximum of two missed cleavages. Precursor and fragment mass tolerance were set to 10 ppm and 0.05 Da, respectively.

GO analysis was performed using the enriched GO procedure in the cluster Profiler R package (PMID: 22455463). Adjusted *P*-values were obtained using the Benjamini–Hochberg method. The proteome enrichment results with or without stress were log_2_ transformed.

### Single-cell migration assays and time-lapse microscopy

To perform the cell migration assay, cells were seeded in wells of a 24-well tissue culture plate at 5 × 10^4^ cells per well in complete medium and were incubated in a cell incubator overnight. In the experiment, cells with or without stress/inhibitors treatment were observed by using Cytation1 (BioTek) under 4× objective, and their cell positions were recorded every 25 min with a total time scale of 12 h. Images were analyzed with ImageJ software and the Chemotaxis and Migration Tool 2.0.

### Wound healing assay

To evaluate the effect of solid stress on cell migration, 2 mL of 5637 cell suspensions were seeded at a concentration of 2 × 10^5^ cells/mL in 12-well plate. Cells were incubated for 24 h to form a confluent monolayer. Then, cells in the middle of the wells were removed by a scratching tip. Cells were washed with PBS for two times to remove cell debris. Cell media free of FBS was added in the wells. Afterward, cell monolayers were treated with or without 3× glass, respectively. Triplicates were made for each group. The wound gap was tracked by Cytation1 Image System (BioTek) for 12 h. Cell migration was evaluated by ImageJ.

### Transwell assay

2 × 10^4^ cells were seeded in the upper chamber (8 μm pore size, Corning) of a 24-well plate with 200 μL complete cell culture medium overnight. On the following day, the medium in the upper chamber was replaced with fresh FBS-free cell medium and covered with 2% agarose gel, while the lower chamber was filled with 500 μL complete medium containing 20% FBS. The chamber was incubated at 37 °C for 24 h. Subsequently, cells were stained with Hoechst (4 μg/mL) at 37 °C for 15 min, followed by detection of migrated cells by confocal microscopy (Olympus). The number of migrated cells was counted by ImageJ software.

### CKAP4 condensation mediated microtubule branching in 3D culture

To establish the spheroid-based 3D model, a low adhesive substrate was produced in advance with 0.05% agarose gel in 96-well plates. Afterward, 400,000 CKAP4-GFP expressing 5637 cells in 100 μL complete cell media were added onto agarose-based substrate overnight. The next day, sparse spheroids of around 50 μm were formed. The size of spheroids was tracked in the following 2 weeks. To investigate CKAP4 phase separation in spheroids, the nucleus was stained by Hoechst for 15 min, then spheroids were gently transferred into a confocal dish for observation.

To investigate CKAP4 phase separation under solid stress in 3D culture, and verify the role of microtubule branching in 3D migration, agarose gel was used to exert solid stress on spheroids. Briefly, 2% agarose solution was prepared by dissolving 0.1 g agarose in 5 mL cell media in micro-oven. After a brief cooling (below 50 °C), fluorescently stained spheroids were gently transferred into 2% agarose solution in confocal dish, and fast cooling in 4 °C for 10 min to avoid the side effect of heat. Afterward, 200 μL complete cell media was added into dish for culture. Nucleus and microtubules were stained by Hoechst and Tubulin Tracker Deep Red, respectively. Spheroids were observed under a 63× confocal microscope (Olympus).

### Mouse studies on cancer metastasis in vivo

Cell suspensions of 5637 shNC and shCKAP4 cells were firstly prepared in FBS-free RPMI-1640 cell medium, respectively. Before injection, 66 μL of cell suspensions were mixed with 33 μL matrixgel on ice to get a final density of 1 × 10^6^ cell/mL. Subcutaneous injections in a final volume of 100 μL were performed in both left and right flank of 6-week-old female NCG mice. When the tumor size reached around 12 mm in diameter, solid stress was applied around the tumor with a rubber ring, labeled as STRESS group. As control, the other 3 mice without rubber ring as NO STRESS group. All mice were fed another 12 weeks, at which time point mice in STRESS group showed reduced activity. Then, mice were sacrificed, organs including heart, liver, spleen, lung, kidney and tumor were collected, fixed and sectioned for analysis.

To further verify the effect of solid stress and the role of CKAP4 phase separation, Losartan, a chemical reagent to release the stress in tumor, was used as reported^55^. Briefly, one week after tumor was stressed with rubber ring, losartan with a concentration of 75 mg/kg was injected twice a week from tail. For control, DMSO solution was injected. All mice were fed another 12 weeks, at which time point mice in the DMSO group showed reduced activity. Mice were sacrificed, organs including heart, liver, spleen, lung, kidney and tumor were collected, fixed and sectioned for analysis.

For H&E staining, Ki-67 and CKAP4 staining, organs were fixed in 4% PFA and embedded in paraffin. Free-floating sections were obtained using a vibratome slicing system. After deparaffinization with various concentrations of ethanol, sections were stained using H&E, Ki-67 and CKAP4 antibodies. Metastatic nodules were counted under a microscope. To verified the effect of CKAP4 condensation, sectioned slices were observed under confocal microscope.

## Supplementary information

Cancer cells sense solid stress to enhance metastasis by CKAP4 phase separation-mediated microtubule branching
