# Supplementary material for: Cancer cells sense solid stress to enhance metastasis by CKAP4 phase separation-mediated microtubule branching
Source: Cell Discov. 2024 Nov 12;10:114. doi: 10.1038/s41421-024-00737-1 (PMC11554681; doi:10.1038/s41421-024-00737-1)
Supplement: Supplementary file 1 — Cancer cells sense solid stress to enhance metastasis by CKAP4 phase separation-mediated microtubule branching [file 41421_2024_737_MOESM1_ESM.pdf]

Supplementary Information

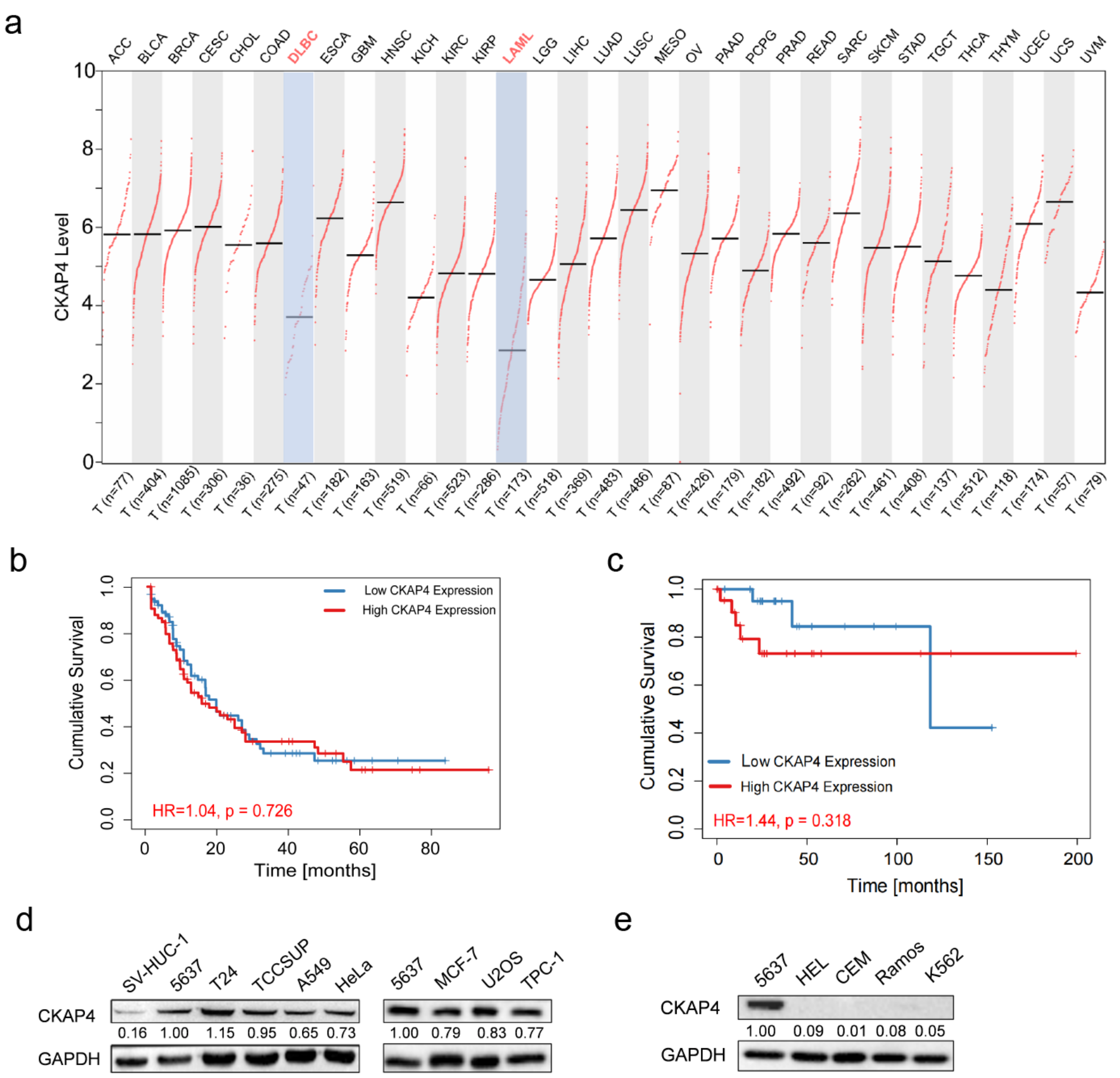

Fig. S1. CKAP4 expression in hematological cancers, such as LAML and DLBC, is lower than that in solid tumors, and it is not a risk factor for survival of LAML and DLBC. a, CKAP4 expression across a broad variety of cancers, of which two hematological cancers, LAML and DLBC, were marked as blue shadow. Data showed transcripts per million from TCGA database. **b**, survival of LAML patients in TCGA database, as stratified by CKAP4 level. P value was calculated by log-rank test. **c,** survival of DLBC patients in TCGA database, stratified by CKAP4 level. P value was calculated by log-rank test. **d**, western blotting quantified CKAP4 expression across a normal bladder epithelial cell line (SV-HUC-1) and a variety of solid tumor cell lines

(human bladder carcinoma, 5637, T24, TCC-SUP; lung adenocarcinoma, A549; human cervical carcinoma, Hela; human breast adenocarcinoma, MCF-7; human bone osteosarcoma, U2OS; human papillary thyroid carcinoma, TPC-1). Relative expression level was marked below. **e,** western blotting quantified CKAP4 expression across a variety of hematological cancer cell lines (T cell leukemia, CEM; human erythromyeloblastoid leukemia, K562; human megakaryocyte, HEL; B lymphocyte, Ramos).

Abbr.: ACC,adrenocortical carcinoma; BLCA, bladder urothelial carcinoma; BRCA, breast invasive carcinoma; CESC, cervical squamous cell carcinoma and endocervical adenocarcinoma; CHOL, cholangiocarcinoma; COAD, colon adenocarcinoma; DLBC, lymphoid neoplasm diffuse large B-cell Lymphoma; ESCA, esophageal carcinoma; GBM, glioma; HNSC, head and neck squamous cell carcinoma; KICH, kidney chromophobe; KIRP, kidney renal papillary cell carcinoma; LAML, acute myeloid leukemia; LGG, brain lower grade glioma; LIHC, liver hepatocellular carcinoma; LUAD, lung adenocarcinoma; LUSC, lung squamous cell carcinoma; MESO, mesothelioma; OV, ovarian serous cystadenocarcinoma; PAAD, pancreatic adenocarcinoma; PCPG, pheochromocytoma and paraganglioma; PRAD, prostate adenocarcinoma; READ, rectum adenocarcinoma; SARC, sarcoma; SKCM, skin cutaneous melanoma; STAD, stomach adenocarcinoma; TGCT, testicular germ cell tumors; THCA, thyroid carcinoma; THYM, thymoma; UCEC, uterine corpus endometrial carcinoma; UCS, uterine carcinosarcoma; UVM, uveal melanoma.

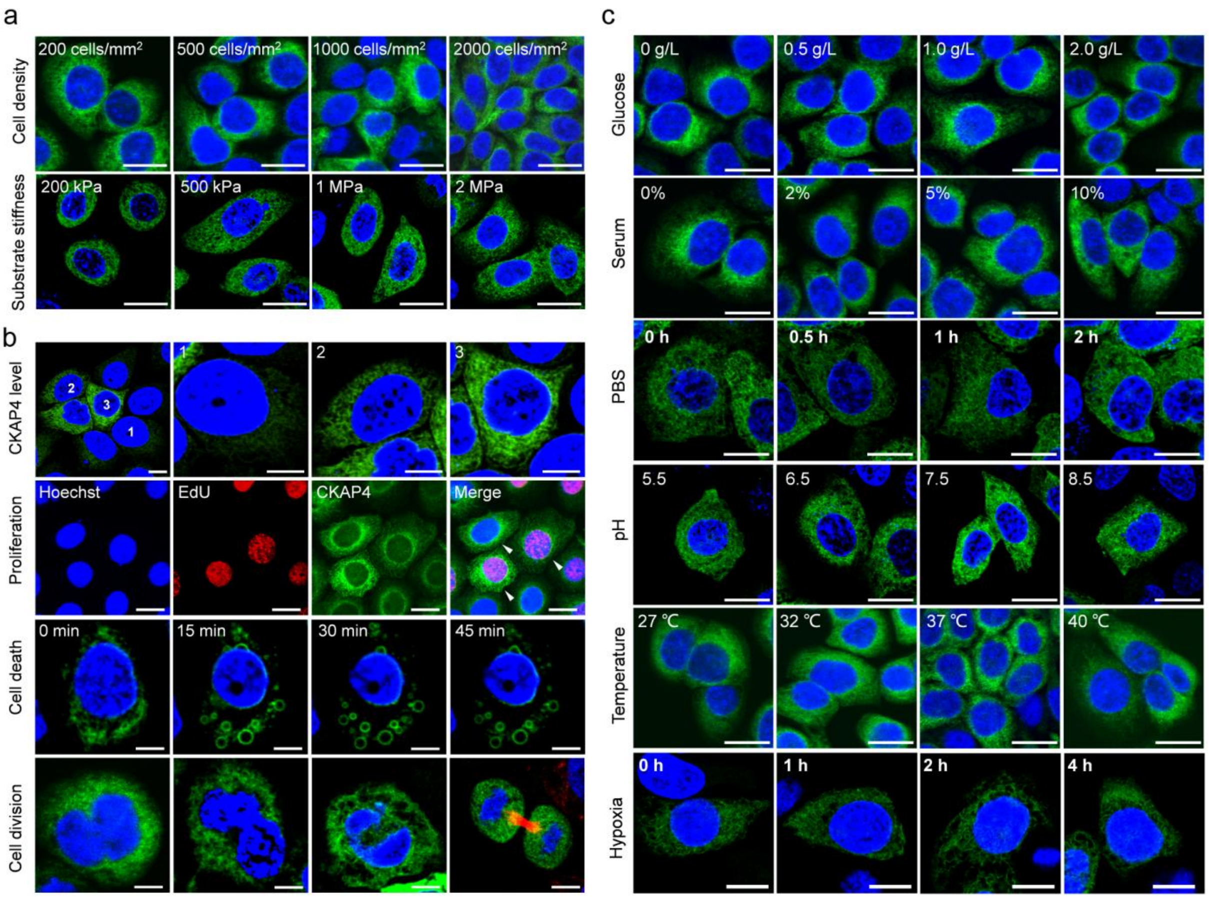

Fig. S2. CKAP4 condensation does not responds to a variety of parameters in TME. a, the effects of cell density and substrate stiffness on CKAP4 condensation. **b**, representative fluorescent images show the effects of CKAP4 expression, cell division, proliferation and death on CKAP4 condensation. **c**, representative fluorescent images show the effects of glucose, serum, nutrition, pH, temperature, and hypoxia on CKAP4 condensation. Scale bar, 10 μm.

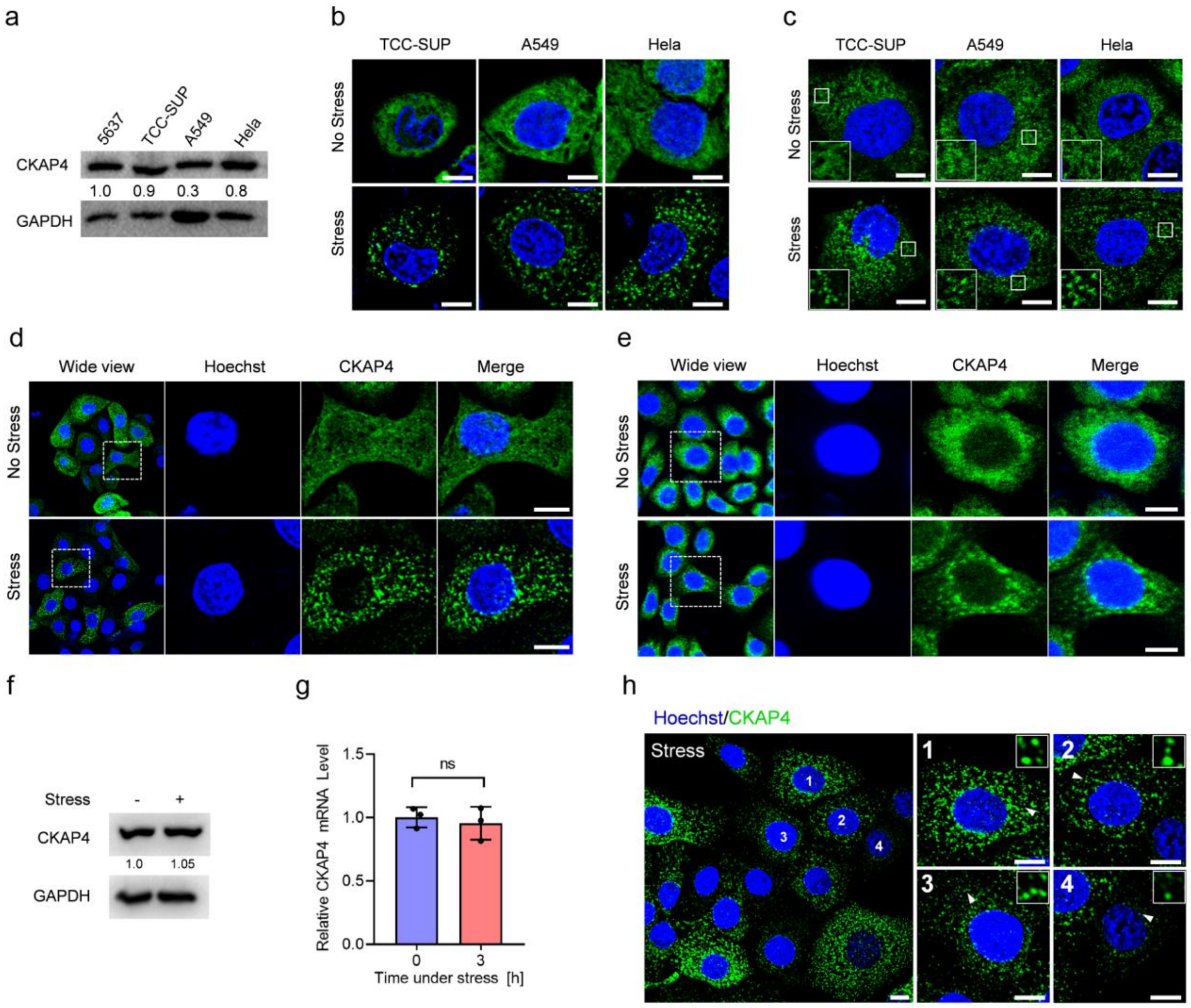

Fig. S3. CKAP4 condensation is commonly observed in a subset of cancer cell lines. a, CKAP4 level in various cancer cell lines identified by western blotting. **b-c**, representative live cell image (b) and IF (c) images of CKAP4 condensation in various cell lines under 3 h of solid stress. **d,** representative images of 5637 cells expressing N terminal GFP-fused CKAP4 with or without solid stress. **e**, representative immunofluorescent images of endogenous CKAP4 in 5637 cells with or without solid stress. **f**, western blotting of CKAP4 expression under 3 h of solid stress. **g**, mRNA level of CKAP4 under 3 h of solid stress, data represent mean ± SD, p value was calculated by Student's t-test. **h**, CKAP4 condensation in cells with different expression levels. Scale bar, 10 μm.

a

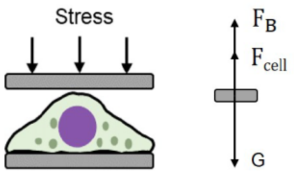

At equilibrium, when the sum of the forces is zero

$$G + F_{cell} + F_B = 0, \quad \dots (1)$$

where,

$$F_{cell} = -N \cdot F \quad \dots (2)$$

$$G = \rho_{glass} \cdot V_{glass} \cdot g = \rho \cdot \pi \cdot r^2 \cdot h \cdot g \quad \dots (3)$$

$$F_B = -\rho_W \cdot V_{glass} \cdot g = \rho_W \cdot \pi \cdot r^2 \cdot h \cdot g \quad \dots (4)$$

Consider the parameters as: radius r=7.5 mm, thickness h=0.15 mm, density ρ=2.5 g/cm^3, cell number N=2.5x10^5, water density ρ_W=1.0 g/cm^3, g=9.8 m/s^2.

Mean force of one piece of glass on one cell: $F=\frac{G+F_B}{N}=\frac{\rho \cdot \pi \cdot r^2 \cdot h \cdot g - \rho_W \cdot \pi \cdot r^2 \cdot h \cdot g}{N}=1.54$ nN

As contact area is a circle with a radius of 5 μm, the pressure: $P=\frac{F}{S}=\frac{F}{\pi \cdot r^2}=19.6$ Pa

Total pressure from 2~6 pieces of glass coverslips: $P_{Total}=(2\sim6) \cdot P=0.039 \sim 0.11$ kPa

b

Tumor type	Solid stress [kPa]
Brain tumors	0.020±0.001
Breast cancer	0.004-0.574
Colorectal tumors	~0.85
Pancreatic tumors	~1

Fig. S4. Calculation and comparison of solid stress in cells and in tumors. a, mechanical model and the calculation of stress from glass coverslips on cells based on classic physical model. **b**, reported levels of solid stress in tumor[4,38,39].

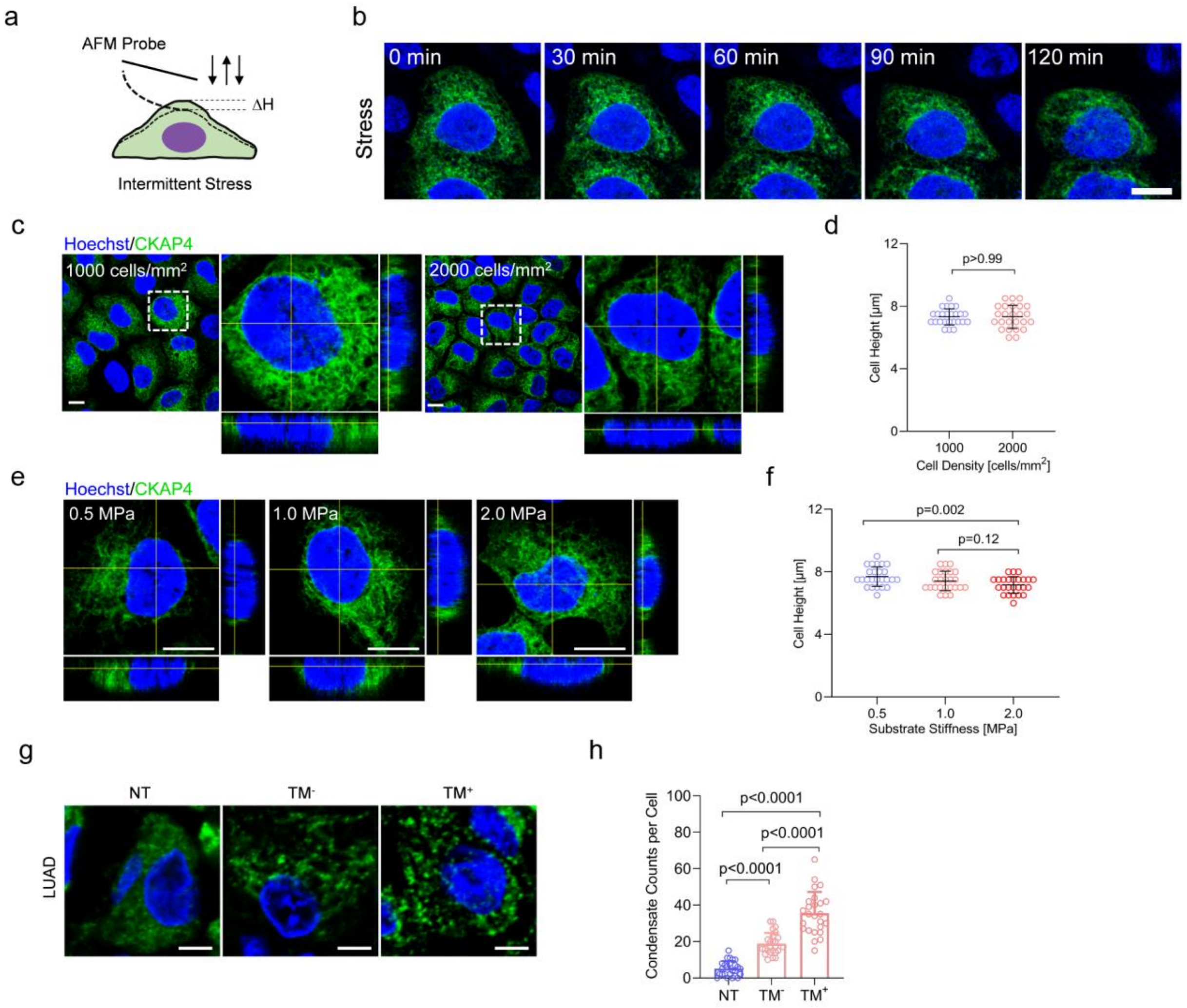

Fig. S5. Proper and continuous solid stress, but not cell density or substrate stiffness, is necessary and sufficient to induce CKAP4 condensation. a, schematic of discontinuous stress on cells. **b**, representative live cell images of CKAP4-GFP in 5637 cells under discontinuous stress of 2 nN. c**,** representative three view confocal images of cells with different cell density. CKAP4 was shown in green, Hoechst in blue. **d**, quantification of cell height in different cell density. **e**, representative three view confocal images of cells seeded on substrate of different stiffness. CKAP4 was shown in green, Hoechst in blue. **f**, quantification of cell height on substrate of different stiffness. **g**, representative images of CKAP4 condensation in tumor tissues of lung cancer patients. **h**, analysis of CKAP4 condensation in NT, TM^- and TM^+ patients of lung cancer. In **d, f, h,** data represent mean ± SD, p value was calculated by Student's t-test. Scale bar, 10 μm.

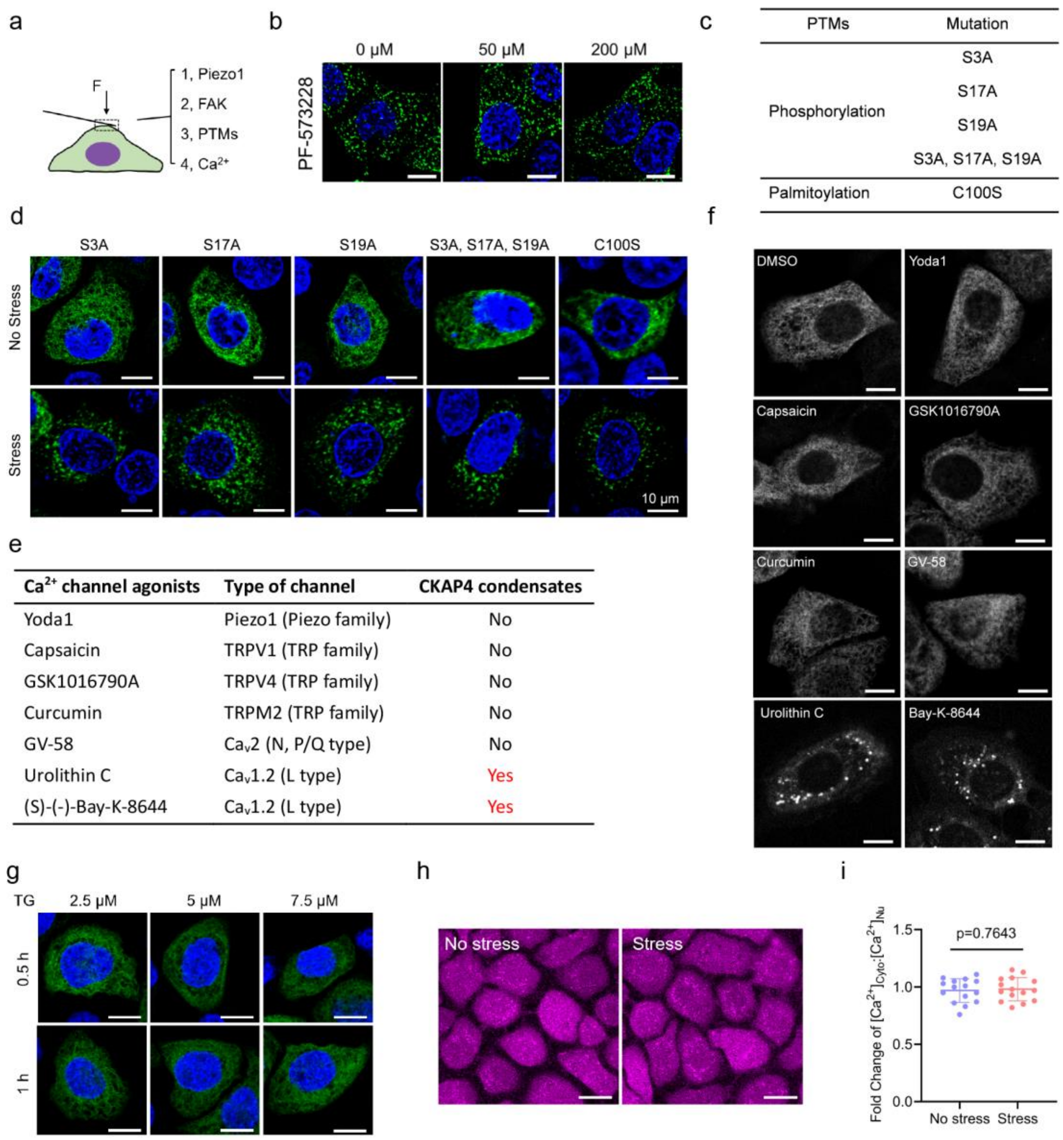

Fig. S6. CKAP4 condensation is dependent on L-type calcium channels, but neither Piezo1, FAK, and PTMs, nor ER- or nucleus-derived calcium. a, mechanical factors that may potentially initiate CKAP4 condensation. **b**, intracellular CKAP4 condensation upon inhibition of FAK by PF-573228 in cells under stress. **c**, reported sites of PTMs in CKAP4. **d**, intracellular CKAP4 condensation upon mutations of PTM sites. **e**, available agonists of calcium channel used in this study. **f**, CKAP4-GFP expressing 5637 cells were treated with 50 μM of calcium channel agonists listed in **e** for 1 h. **g**, intracellular CKAP4 condensation upon TG treatment. **h**, intracellular Ca^{2+} change indicated by Cal-590 under solid stress. **i**, quantification of intracellular Ca^{2+} change between cytosol and nucleus. Data represent mean ± SD, p value was calculated by Student's t-test. Scale bar, 10 μm.

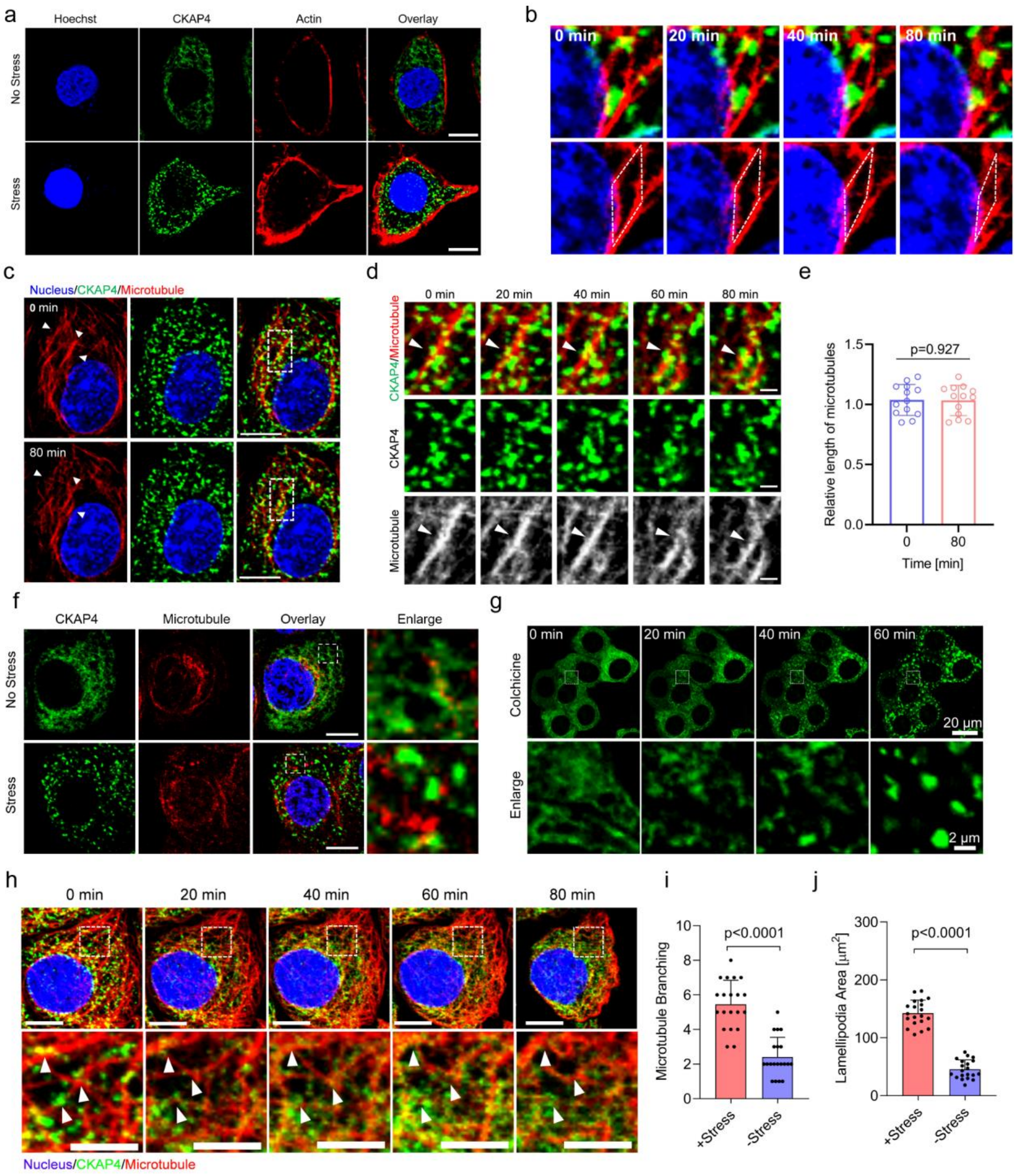

Fig. S7. CKAP4 serves as a mechano-regulator modulating microtubule branching. a, solid stress-induced CKAP4 condensation and the formation of peripheral actin. Scale bar, 10 μm. **b**, CKAP4 condensation tuned the degree of microtubule intersection. Nucleus, blue; CKAP4, green; microtubule, red. **c**, CKAP4 condensates regulated microtubule branching in the timepoint of 0 and 80 min. Nucleus, blue; CKAP4, green; microtubule, red. Scale bar, 10 μm. **d**, time lapse imaging of CKAP4 condensates regulated microtubule branching in the indicated spot

of **c**. CKAP4, green; microtubule, red. White arrows indicated the microtubule under branching. Scale bar, 2 μm. **e**, quantification of overall length of microtubules in cells under solid stress. **f,** CKAP4 condensation in cells pretreated with Albendazole (inhibitor of microtubule polymerization). Scale bar, 20 μm. **g**, CKAP4 condensation in cells pretreated with colchicine (inhibitor of microtubule formation). CKAP4 was shown in green color. **h**, microtubule and CKAP4 change after the release of external solid stress. Scale bar, 10 μm. **i**, microtubule branching change after the release of solid stress. More than 20 cells were calculated for analysis. **j**, lamellipodia change after the release of solid stress. More than 20 cells were calculated for analysis. In **e, g, i,** and **j**, data represent mean ± SD, p value was calculated by Student's t-test.

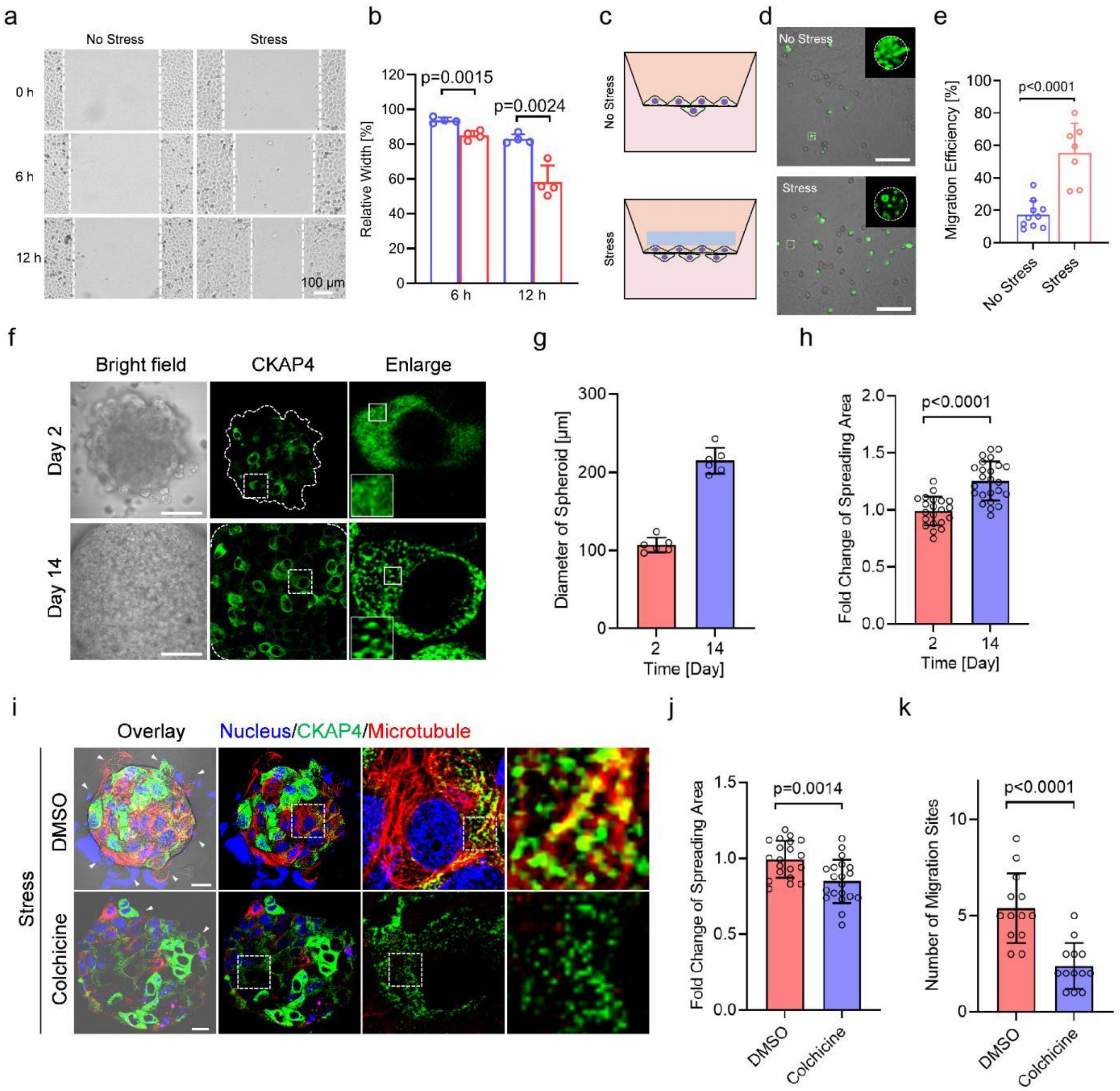

Fig. S8. Microtubule branching promotes cell migration in 3D culture. a, wound healing of 5637cells in response to solid stress. **b**, wound gaps were calculated in more than three individual experiments. **c**, schematic of cells in transwell migration assay. **d**, representative images of transwell migration assay of CKAP4-GFP-expressing 5637 cells treated with (right panel) and without (left panel) solid stress. CKAP4 condensation in the well was indicated in the pores. Scale bar, 100 μm. **e**, analysis of migrated cells in **d**. **f**, CKAP4 underwent phase separation as solid stress accumulated within spheroid. Scale bar, 50 μm. **g**, diameter of spheroids as the growth of spheroids. **h**, fold change of spreading area of cancer cells within spheroids. More than 20 cells were calculated. **i**, CKAP4 condensation-driven microtubule branching within spheroid

under solid stress. Spheroids were treated with either DMSO or colchicine. White arrows indicated the migration sites on the surface of spheroids. Scale bar, 20 μm. **j**, fold change of spreading area of cells within spheroids. **k**, number of migration site on the surface of spheroids. In **b**, **e**, **h**, **j**, **k**, data represent mean ± SD, and p value was calculated by Student's t-test

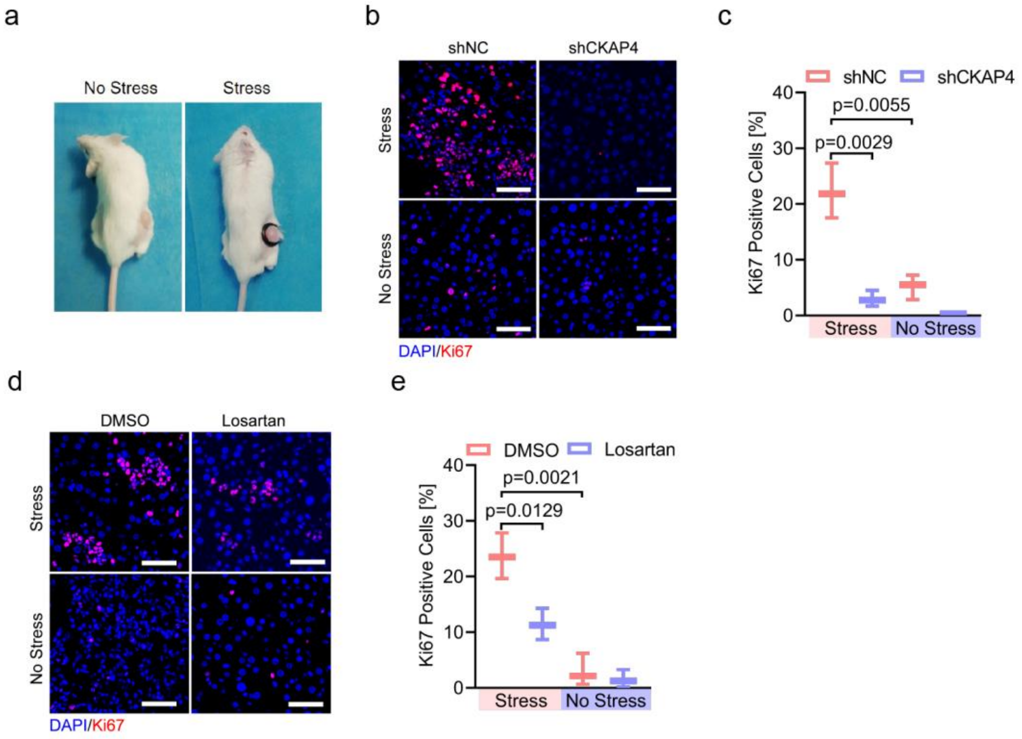

Fig. S9. CKAP4 condensation mediates solid stress-induced cancer metastasis. **a**, representative images of in vivo study to investigate the effect of solid stress. **b**, representative images of metastatic nodules of shNC or shCKAP4 5637 cancer cells in liver was determined by ki67 staining. **c**, quantification of metastatic nodules in **b**. **d**, representative images of metastatic nodules of 5637 cancer cells in liver was determined by H&E staining. Losartan was intravenously injected to release the stress in tumor sites. **e**, quantification of metastatic nodules in **d**. In **c**, **e**, data represent mean ± SD, and p value was calculated by Student's t-test. Scale bar, 50 μm.
